# Biomimetic Convex Implant for Corneal Regeneration Through 3D Printing

**DOI:** 10.1002/advs.202205878

**Published:** 2023-02-12

**Authors:** Yingni Xu, Jia Liu, Wenjing Song, Qianchun Wang, Xiaomin Sun, Qi Zhao, Yongrui Huang, Haochen Li, Yuehai Peng, Jin Yuan, Baohua Ji, Li Ren

**Affiliations:** ^1^ School of Materials Science and Engineering National Engineering Research Center for Tissue Restoration and Reconstruction Key Laboratory of Biomedical Engineering of Guangdong Province Key Laboratory of Biomedical Materials and Engineering of the Ministry of Education Innovation Center for Tissue Restoration and Reconstruction South China University of Technology Guangzhou 510006 P. R. China; ^2^ Wenzhou Institute University of Chinese Academy of Sciences Wenzhou 325001 P. R. China; ^3^ National Engineering Research Center for Tissue Restoration and Reconstruction Key Laboratory of Biomedical Engineering of Guangdong Province Key Laboratory of Biomedical Materials and Engineering of the Ministry of Education Innovation Center for Tissue Restoration and Reconstruction South China University of Technology Guangzhou 510006 P. R. China; ^4^ Guangzhou Proud Seeing Biotechnology Co., Ltd Guangzhou 510320 P. R. China; ^5^ State Key Laboratory of Ophthalmology Zhongshan Ophthalmic Center Sun Yat‐sen University Guangzhou 510623 P. R. China; ^6^ Institute of Biomechanics and Applications, Department of Engineering Mechanics Zhejiang University Hangzhou 310027 P. R. China; ^7^ Bioland Laboratory Guangzhou Regenerative Medicine and Health Guangdong Laboratory Guangzhou 510005 P. R. China

**Keywords:** 3D printing, slope gradient, cell adhesion, cellular mechanosensing, corneal regeneration

## Abstract

Blindness caused by corneal damage affects millions of people worldwide, and this number continues to rise. However, rapid epithelization and a stable epithelium process are the two biggest challenges for traditional corneal materials. These processes are related to corneal curvature, which is an important factor in determination of the corneal healing process and epithelial behavior during corneal damage. In this study, smooth 3D‐printed convex corneal implants based on gelatin methacrylate and collagen are generated. As epithelium distribution and adhesion vary in different regions of the natural cornea, this work separates the surfaces into four regions and studies how cells sense topological cues on curvature. It is found that rabbit corneal epithelial cells (RCECs) seeded on steeper slope gradient surfaces on convex structures result in more aligned cell organization and tighter cell‐substrate adhesion, which can also be verified through finite element simulation and signaling pathway analysis. In vivo transplantation of convex implants result in a better fit with adjacent tissue and stronger cell adhesion than flat implants, thereby accelerating corneal epithelialization and promoting collagen fibers and neural regeneration within 180 days. Taken together, printed convex corneal implants that facilitate corneal regeneration may offer a translational strategy for the treatment of corneal damage.

## Graphical abstract

A smooth surface corneal material with curvature is prepared by 3D printing to promote corneal stroma regeneration and corneal function recovery. The results show convex cornea can induce cell alignment and promote epithelial adhesion, making repair faster and more firmly. 3D printing accompanied with mechanism research provide a new idea for the design of corneal repair materials.

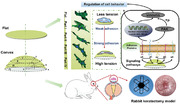

## 1 Introduction

Blindness caused by corneal damage ranks second in the ophthalmology field, with approximately 12 million people worldwide with various corneal diseases reported every year.^[^ ^1^ ^]^ Owing to the prolonged use of digital devices, approximately 23.7% of the population suffers from dry eye,^[^ ^2^ ^]^ which can cause blindness if severe.^[^ ^3^ ^]^ The market demand to solve blindness, therefore, has ample potential. Lamellar and penetrating keratoplasty of allogeneic cornea is currently the only treatment for visual impairment, but it is limited by a shortage of donor corneal tissue. Thus, there is a pressing clinical need to create corneal substitutes. To date, a number of corneal materials have been developed based on natural^[^ ^1^, ^4^, ^5^ ^]^ and synthetic materials^[^ ^6^, ^7^, ^8^ ^]^ or their combination. Despite the fact that they mimic native cornea in terms of components and biochemical cues, disadvantages such as epithelization difficulties, epithelium‐off problems,^[^ ^9^ ^]^ weak mechanical properties under suture and corneal dissolution^[^ ^7^ ^]^ limit their applications in clinical trials, among which fast epithelization and epithelium stability are two challenges that have remained elusive for corneal regeneration. Strategies to overcome these difficulties should first consider typical corneal curvature structures based on the principle that structure determines functions.

The cornea is an anisotropic structure with curvature, and it has been reported that the mean cohesive strength in the inferior periphery differs from the strength of the superior periphery.^[^ ^10^ ^]^ Anisotropy is thought to be a crucial determinant of corneal shape during ectatic disease. Epithelial behavior is related to corneal biomechanics,^[^ ^11^ ^]^ illustrating that the relationship between curvature structure and epithelial behavior is pivotal in guiding corneal regeneration. Recently, there has been increasing evidence that environmental signaling to cells entails much more than biochemical cues.^[^ ^12^ ^]^ Curvature, which is one of the most common topographies in vivo, can affect cell fate during development and is closely associated with diseases such as cancer.^[^ ^13^ ^]^ Pioneering studies^[^ ^14^ ^]^ using polydimethylsiloxane (PDMS) based topographies showed that curvature can affect cell shape, alignment, polarization, and differentiation of chondrocytes and myoblasts. Despite efforts to understand the impact of curvature on cell behavior, the substrates used were mostly resins that were suitable for structural research but that did not exhibit excellent biocompatibility. In addition, these studies focused on nano‐ or micron‐scale surfaces, and the tissue‐size (milliscale) curvature was largely overlooked and underestimated. Whether the curvature of corneal implants can influence epithelial behavior and further induce corneal regeneration remains unknown.

Corneal curvature can determine the corneal healing process and epithelial behavior. Hyperplasia of the epithelium occurs when the curvature is too steep or on flat surfaces to compensate for primary epithelium loss, and increased epithelium thickness may result in corneal instability.^[^ ^15^ ^]^ In addition, epithelium is closely related to the formation of tear film, and they are interconnected, with normal tear film being a major prerequisite for corneal reconstruction.^[^ ^16^ ^]^ Numerical simulations have shown that corneal curvature can influence tear film dynamics^[^ ^17^ ^]^ and shear‐thinning properties,^[^ ^18^ ^]^ curvature delays the risk of tear film rupture compared to a flat substrate, and the distribution of tear film is more uniform on curved surfaces, which can stabilize epithelium growth and adhesion, thus promoting corneal regeneration. In addition, it is estimated that cornea materials with curvature should fit well with neighboring tissue, which reduces the number of sutures required and hence decreases the mechanical demands on cornea materials. Curvature is a typical biomechanical cue of the cornea and plays a fundamental role in guiding epithelium recovery and corneal regeneration. Generating curvature and illustrating the relationship between cell behavior and curvature of corneal implants is essential. As an additive manufacturing technology, 3D printing has attracted significant attention for the fabrication of complex tissues, owing to its cost‐efficient production and personalized customization. Only a few studies^[^ ^5^, ^19^ ^]^ have been conducted to date to print cornea owing to high requirements; although they mimicked either curvature or orthogonally aligned structures, problems relating to a lack of practical application remain because of weak mechanical properties, opacity, thickness, and step‐effect surface. Even one study established^[^ ^19a^ ^]^ animal lamellar keratoplasty, it has the drawback of a slow and unstable epithelization process. Therefore, developing a 3D‐printed corneal implant with smooth curvature and possessing functions of mediating epithelium behavior are of clinical significance.

To develop an ideal curved corneal implant similar to the native cornea appropriate for clinical application, we fabricated a convex corneal implant with a smooth surface through 3D printing that provided a basis for mechanistic and in vivo studies. In particular, we prepared ink with a rapid temperature transition based on gelatin methacrylate (GelMA)^[^ ^20^ ^]^ and collagen type I^[^ ^21^ ^]^ for which the extruding state was the premise for acquiring convex implants with smooth surfaces. The resulting implants possessed curved structures that could guide cell organization and adhesion. First, as the printed implant was a dome‐shaped cornea and cells tended to react to topographical cues,^[^ ^22^ ^]^ we sought to document how the slope gradient of the convex structure mediated cell behavior by cytoskeleton/nuclei analysis, and a finite element method (FEM) simulation^[^ ^23^ ^]^ was constructed to reveal dynamic interaction between cell tension and adhesion of cell‐substrate, and a signaling pathway was also proposed to analyze adhesion behavior. Second, we showed that the superior convex structures could promote epithelialization, cell adhesion, and neuron regeneration to heal corneal defects after transplantation of printed convex implants into a rabbit partial keratectomy corneal injury model within 180 days of observation, which opens new avenues for material design. A mechanism by which convex corneas promote corneal regeneration is proposed.

## 2 Results

### 2.1 Suppression of Step Effect for Printing of Convex Cornea Implants

**Figure 1:**
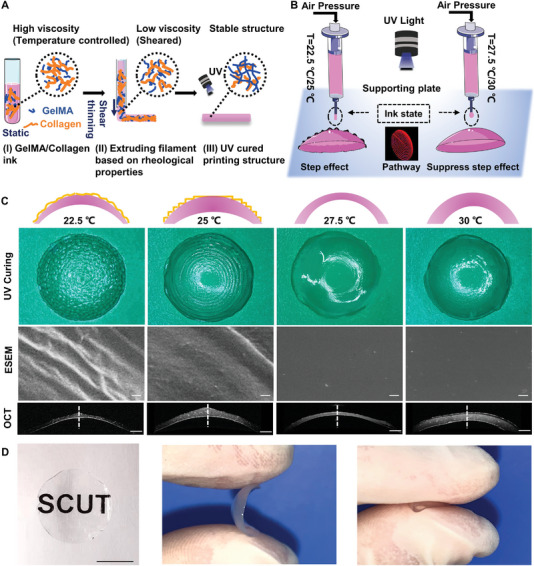
Fabrication of 3D‐printed convex cornea and strategy of suppressing step effect. A) Schematic illustration of printing implants with GelMA/Collagen ink. B) Schematic illustration of the temperature‐controlled printing process to suppress step effect. C) Images of ink deposition at different extruding temperatures and further surface details of printed cornea implants. Bright‐field images in UV Curing displayed the actual structure of printed cornea, scale bar = 200 µm. Environmental scanning electron microscope (ESEM) revealed microsurface, scale bar = 200 nm. OCT exhibited images of cornea printed at different temperatures, scale bar = 1 mm. D) Digital photos of 3D‐printed cornea implants showing material transparency and flexibility.

Temperature‐sensitive inks have been widely used in the 3D printing field owing to their shear‐thinning and rapid sol–gel transition. Here, a UV‐curable hydrogel system with temperature sensitivity was designed for the corneal implant to ensure successful printing, which includes GelMA (Figure S1, Supporting Information), collagen, and the photoinitiator 2‐hydroxy‐4′‐(2‐hydroxyethoxy)‐2‐methylpropiophenone (I2959, 0.5% (w/v) final concentration), and their shear‐thinning and rapid sol–gel transition are displayed in Figure S2 (Supporting Information) to achieve successful printing. The 3D printing was based on temperature control and printing pathways, as shown in **Figure** **1**A,B. With traditional printing parameters of 22.5 °C or 25 °C, the printed cornea had a serious step effect, resembling a whorl, as shown in environmental scanning electron microscope (ESEM) images (Figure 1C). When the extrusion temperature was 27.5 °C or 30 °C, the droplets tended to stream slightly outwards and downwards along the printing pathway, resulting in the center of the implant being thinner and the periphery being thicker, which was nearly the same as the natural corneal structure. Owing to the near‐sol state of the deposited inks, the casting phenomenon generated a smooth surface, as shown in ESEM images and Figure S4A (Supporting Information). All implants had curvature structures with different thickness characteristics (OCT sections, Figure S6D, Supporting Information). Temperatures below 25 °C produced implants with a thick tip, and when the temperature was increased to 25 °C, the tip effect was more apparent up to 625 µm. A temperature of 27.5 °C produced the most similar structures as natural cornea, with an average thickness of 186 µm. Based on the high requirements of transparency and mechanical properties (Figure S7, Supporting Information), the resultant ratio of GelMA/collagen inks was 10% to 0.6%. The printed implants with porous structures (Figure S7A, Supporting Information), light transmittance of 83% at a wavelength of 750 nm, tensile strength of 51 kPa, compressive modulus of 130 kPa, water content of 83%, and mass enzymatic resistance were most similar to native corneal tissue. They could readily be operated and were suitable for our subsequent experiments (Figure S6G, Supporting Information).

### 2.2 Slope Gradient Modulates Rabbit Corneal Epithelial Cell (RCEC) Morphology and Orientation

**Figure 2:**
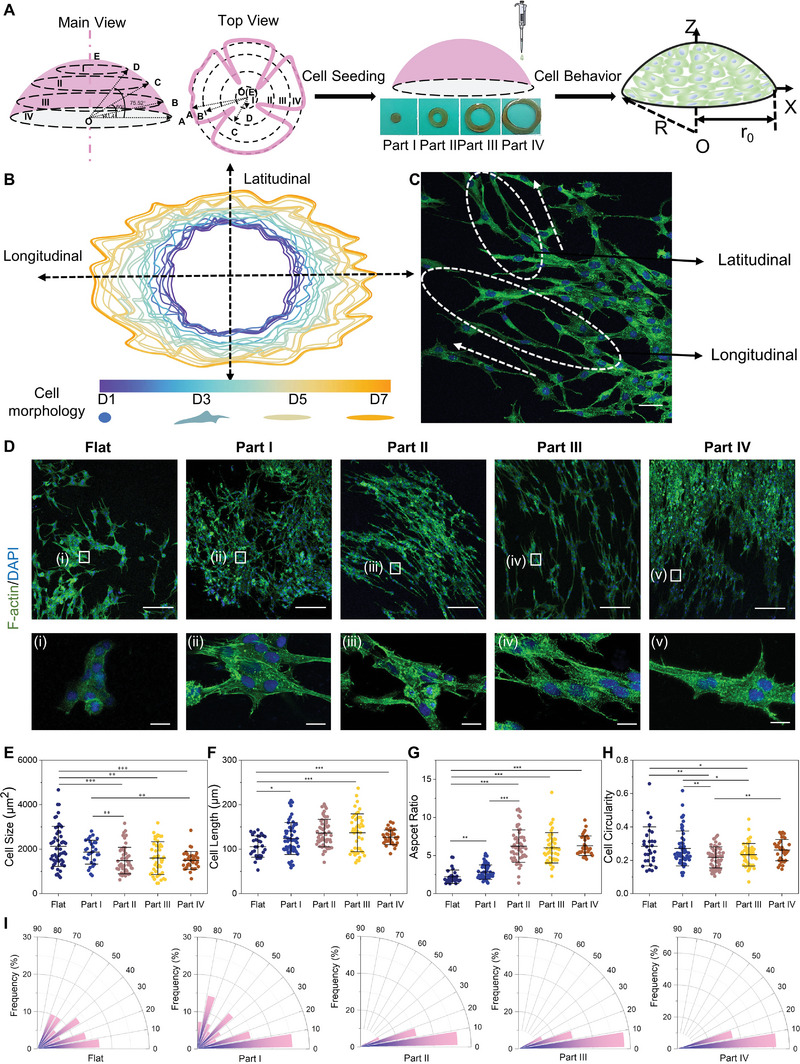
Substrate slope gradient induced rabbit corneal epithelial cell (RCEC) morphology and alignment behavior. A) Schematic illustration of how to observe cell behavior. B) Schematic diagram of cell trajectories during 7 days of growth on 3D‐printed convex implants, and cell morphology changes with time elapsed at days 1, 3, 5, and 7. Note that cells initially starting at the latitudinal axis turned toward the longitudinal axis as last. C) Representative confocal image at day 7 of RCECs on printed cornea, the image shows cells stretched along the longitudinal axis (marked with a white circle) and cells crawled toward the latitudinal axis (marked with a white circle) until stretched along the longitudinal axis. D) Representative confocal images of RCECs cultured on slope gradient surface for 5 days. Scale bar = 250 µm. i–v) are magnified images, scale bar = 25 µm. Impact of surface slope gradient on RCEC morphology including cell size (E), cell length (F), aspect ratio (G), and cell circularity (H) in (D). All the results were calculated as mean ± standard deviation (SD) (cells were from three independent experiments, *n* = 40). I) Orientation analysis of RCECs from (D), orientation vectors were quantified and grouped into nine intervals every 10° within a 0°–90° range, and the frequency at any interval was then calculated (cells were from three independent experiments, *n* = 120), cells were considered aligned if the angle between the long axis and the grating was <10°.

When rabbit corneal epithelial cells (RCECs) were inoculated on convex constructs, an interesting cell growth phenomenon was observed. Cells proliferated more slowly on convex constructs than on flat constructs (Figure S9B, Supporting Information). As time elapsed, the cells tended to proliferate in the longitudinal direction and grew into an elliptical shape owing to different proliferation rates (Figure S10, Supporting Information). Cells were seen to stretch pseudopodia upwards in the latitudinal direction, indicating the proliferation toward latitude happened by “climbing” (**Figure** **2**B,C).

Previous research has shown that cell organelles, such as the nucleus and centrosome, can orient themselves in response to external physical stimuli.^[^ ^24^ ^]^ Here, the alignment of cells was also observed, which surprised us because previous studies have speculated that curvature radii that are too large cannot be detected by individual cells.^[^ ^25^ ^]^ However, as the curvature was on a milliscale, cell alignment on different slope gradients was still observed. To further investigate the influence of slope gradient on cell behavior, we segmented the convex structure into four parts, as shown in Figure 2A, according to the method in the ophthalmology field.^[^ ^26^ ^]^ After culturing for 5 days, most of the F‐actin of RCECs on convex constructs was aligned along the longitudinal axis. Cells on the convex surface significantly changed their cytoskeleton orientations compared with those on the flat surface (Figure 2D). When the slope gradient increased from flat to Part IV, the cell area gradually decreased and the shape of the cells became more spindle‐like (Figure 2E–H). There were no obvious shape differences between Part II and Part IV, but there was a big change for Part I, which may be because the zone of each part was small and Part I was situated on the top of the convex structure having a mild slope akin to flat. Cells positioned themselves on slope gradient with alignment (Figure 2I), and a steeper slope gradient would stimulate more alignment, whereas cells on the flat implants lacked any specific orientations. The alignment distribution of flat and Part I was dispersive, while the others were much more intense at 0°–20°, and the orientation tended to comply with the cell shape, demonstrating slope gradient effects on regulation of the cytoskeleton.

### 2.3 Slope Gradient Promotes Cell Adhesion and Chromatin Condensation

**Figure 3:**
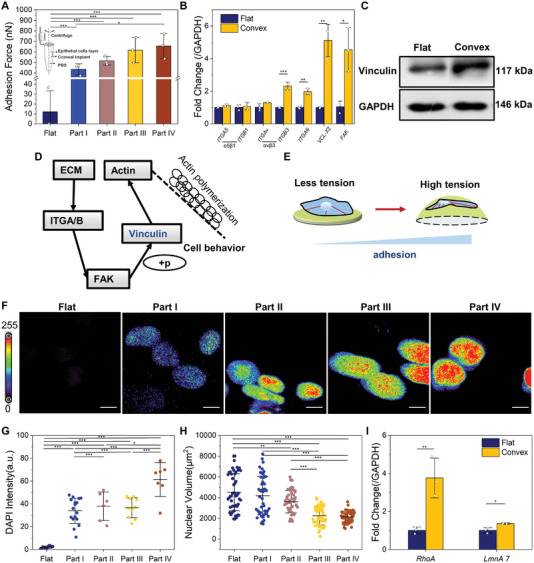
Rabbit corneal epithelial cells (RCECs) sensed interfacial slope gradient through focal adhesion (FA) organization and nuclear deformation. A) RCEC adhesion force using a centrifugation method after being cultured on different parts on day 5. B) qPCR analysis showing that a convex construct can promote the expression of various genes related to adhesion when RECEs were seeded at day 5. C) Western blot result of vinculin expression. D) Schematic diagram of signaling pathway prediction based on the PCR and WB results. E) Schematic illustrating cell tension on substrate. F) Confocal imaging showing enlarged pictures of RCECs stained with DAPI nuclear staining on flat and convex surfaces (scale bar = 10 µm). G) Cells on convex structures exhibited a greater degree of chromatin deformation based on quantification of DAPI intensity. H) Nuclear volume. I) Elevated expression of *RhoA* and *LmnA 7* genes verified deformation of nuclei on a convex construct (cells were from three independent experiments, *n* = 50). Error bars in (A,B), (G–I) represented the standard deviation from the mean.

As epithelial adhesion is a key factor in corneal regeneration, and as GelMA contains many amino acid sequences (RGDs) that can promote cell adhesion, we studied the adhesion behavior of vinculin protein to determine any correlation with the printed topography. RCECs expressed more focal adhesion (FAs) with the slope gradient (Figure S11, Supporting Information). We also calculated the adhesion force on different parts according to the method suggested in the study by Reyes and Garcia,^[^ ^27^ ^]^ and the adhesion force of flat implants was 36.5 nN, while convex structures processed an average adhesion force of 555.8 nN (**Figure** **3**A). Convex structures can promote adhesion force, and the adhesion behavior varies for different slopes, with a steeper slope resulting in a stronger adhesion force. To further investigate the mechanism causing adhesion differences, we investigated the changes in mRNA expression (Figure 3B) and protein levels (Figure 3C) semiquantitatively. The integrin family, a type of cell surface receptor that mediates cell adhesion to extracellular proteins, can bind extracellular ligands to form FAs that participate in both the signaling and mechanical transition crucial to the adhesion process.^[^ ^28^ ^]^ We selected the integrin family comprising integrin *α*v*β*3 and *α*5*β*1; the former supported a persistent lamellipodial mode of static migration, whereas cells adhering to the latter did not maintain polarity and migrated randomly in a dynamic mode.^[^ ^29^ ^]^ As a large number of cells were needed in this experiment, it was a challenge to collect cells from different parts; therefore, we decided to regard cells on convex constructs as a whole in order to distinguish the dissimilitude of flat versus convex implants. It can be seen that while there was no significant difference between integrin *α*5*β*1 there was higher gene expression of integrin *α*v*β*3. In addition, the expression of integrin *α*6 (*ITGA6*), vinculin (*VCL‐X2*), and focal adhesion kinase (*FAK*) mRNAs was enhanced compared to those of flat implants, indicating that the expression of these genes was related to adhesion and static migration. In particular, *VCL‐X2*, which is related to the protein vinculin, increased approximately five times. Furthermore, Western blot experiments were conducted to verify whether vinculin proteins on convex curvature were highly expressed, and the results confirmed this (Figure 3C). These results suggest that not only did convex implants promote cell adhesion behavior compared to flat implants, but the slope gradient of convex curvature also played an infusive role in regulating cell adhesion force, which is a pioneering and interesting discovery.

There is evidence in support of bidirectional mechanical signaling between the cytoskeleton and nucleus.^[^ ^30^ ^]^ Applying forces to the cell membrane causes deformation of the nucleus, confirming that tension is transmitted to nuclei through the cytoskeleton. Since RCECs behaved differently in terms of the shape of the cytoskeleton and adhesion ability on convex constructs, we next investigated how convex curvature affects their nuclei. A quantitative procedure based on DAPI staining was used to investigate the effect of topographical cues on nuclei behavior,^[^ ^12b^ ^]^ and DAPI uptake levels reflected total DNA and chromatin condensation levels,^[^ ^31^ ^]^ according to which, the average spatial density corresponding to the ratio between the integrated fluorescence intensity and the volume of the nucleus is a reliable indicator of the average chromatin condensation.^[^ ^32^ ^]^ It can be seen that fluorescence intensity increased in proportion to the level of chromatin condensation,^[^ ^12c^ ^]^ and the DAPI intensity of cells on flat implants was the lowest (10.2 a.u.), while the DAPI staining of cells on convex curvature was more than 10 times higher, and there was no significant difference between Part II and Part IV, which also corresponded with the adhesion behavior (Figure 3F,G). Marked reorganization of chromatin condensation correlated with deformation of the nuclei (Figure 3H, Movie S2–S3, Supporting Information), and highly condensed chromatin domains exhibited higher fluorescence intensity with decreased volume. As the cells elongated from flat to Part IV regions, the volume of the nuclei decreased, and chromatin condensation tended to start from the periphery toward the center. To further confirm these results, we analyzed nuclear mRNA changes at the molecular level. We then examined Ras homolog family member A (*RhoA* ^[^ ^33^ ^]^) and lamin A (*LmnA 7* ^[^ ^34^ ^]^) genes because *RhoA* is central to mechanotransduction because of its crucial role in regulating the actin cytoskeleton and response to mechanical forces, and lamin A gene expression is related to karyotheca integrity. It was not surprising that cells on convex constructs all exhibited higher expression of these genes than those on flat constructs, indicating a fundamental change in RCECs had taken place (Figure 3I). From the above results, we observed that deformation of the nucleus and chromatin condensation were connected with different cell behaviors on slope gradients and confirmed the role of the nucleus in regulating RCEC shape and adhesion.

### 2.4 FEM Analysis of Cell Tension and Adhesion Can Explain the Observed Cell Organization

**Figure 4:**
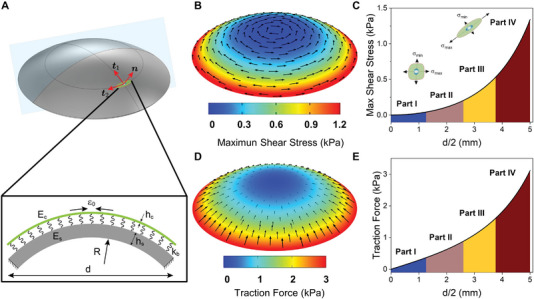
Finite element model (FEM) simulation of the in‐plane stresses and cell‐substrate interaction force (traction force) in the tangent plane of a cell layer. A) FEM of the cell layer and cornea substrate system. The local coordinate system of the cell layer was defined by (*t*_1_,*t*_2_,*n*), representing two tangential directions (*t*_1_ and *t*_2_) and one normal direction (*n*). The cornea substrate and the cell layer were modeled as isotropic solid and prestrained membrane, respectively, where the cell layer was anchored on the cornea substrate via molecular bonds (treated as elastic springs *k*_b_), and *E*_c_ or *E*_s_ is Young's modulus of a prestrained elastic membrane or elastic substrate, respectively. *h*_s_ and *h*_c_ are the thickness of the cornea substrate and cell layer, respectively. *ε*_0_ represented the initial strain, *d* and *R* are the diameter and spherical radius of the cornea model, respectively. B) The in‐plane stresses in the tangent plane of the cell layer. The color map illustrates the maximum shear stress in the tangent plane of the cell layer, and the black arrows represent the direction of the in‐plane maximum principal stress. C) Plot depicting the theoretical predictions for the relationship of maximum shear stress and slope gradient. The insets illustrate the cell polarization and alignment driven by the maximum shear stress: when the two principal stresses were isotropic, the cells were nonpolarized with random orientations; otherwise, the cells were polarized and well‐aligned when the principal stresses were anisotropic. D) The traction force in the tangent plane of the cell layer. The color map and black arrows illustrated the magnitude and direction of traction force in the tangent plane of the cell layer, respectively. E) Plot depicting the theoretical predictions for the relationship of traction force and slope gradient.

In order to gain a better understanding of the effect of surface topography on cell organization and thus provide unique cues for the collective cell behaviors,^[^ ^35^ ^]^ we then adopted the FEM simulation based on COMSOL software to analyze the observed cell self‐organization behaviors. We described the cell cytoskeletal tension mechanics using a spring potential with a stiffness of *k*_b_, which can act as an adhesion molecule linking cell layers to the substrate (full description of Figure S12, Supporting Information). It has been shown that the maximum shear stress drives cell polarization and alignment, and the cell polarization degree, i.e., the cell aspect ratio, is proportional to the maximum shear stress.^[^ ^36^ ^]^ FEM simulation showed that the maximum principal stress of a cell layer was along the circumferential direction (i.e., the *t*_2_ axis) (**Figure** **4**B). The anisotropy of the principal stresses, characterized by the maximum shear stress, varied from isotropic (small maximum shear stress) in Part I to anisotropic (large maximum shear stress) in Part IV (Figure 4C). These results predict that cells are directionally polarized and aligned along the direction of *t*_2_ in Part IV with a large aspect ratio, whereas they are randomly polarized and aligned in Part I with a relatively small aspect ratio, which is consistent with the experimental observation of the cell orientation and aspect ratio. The cell–substrate interaction force (traction force) is generated by the self‐contractility of the cytoskeleton and is transmitted to the substrate by FA. Thus, the distribution of the traction force is closely correlated with the distribution of the size or fluorescence intensity of FAs.^[^ ^37^ ^]^ Our simulation showed that the cell–substrate traction force in the tangent plane increased from Part I to Part IV (Figure 4D,E). Correspondingly, in the experiment, we found that the fluorescence intensity of FAs had the same distribution as the predicted traction force (Figure S11, Supporting Information). Taken together, our FEM simulation predicted the intercellular in‐plane stresses and cell–substrate interaction force of a cell layer adhering to the corneal substrate. Due to the coupling between the chemistry/structure and the mechanics at the cellular level, cells respond to intercellular stresses through the polarization and alignment of the cell/cytoskeleton, which in turn changes the stress state in the cell layer.

### 2.5 Printed Convex Cornea Promotes Rabbit Epithelization

**Figure 5:**
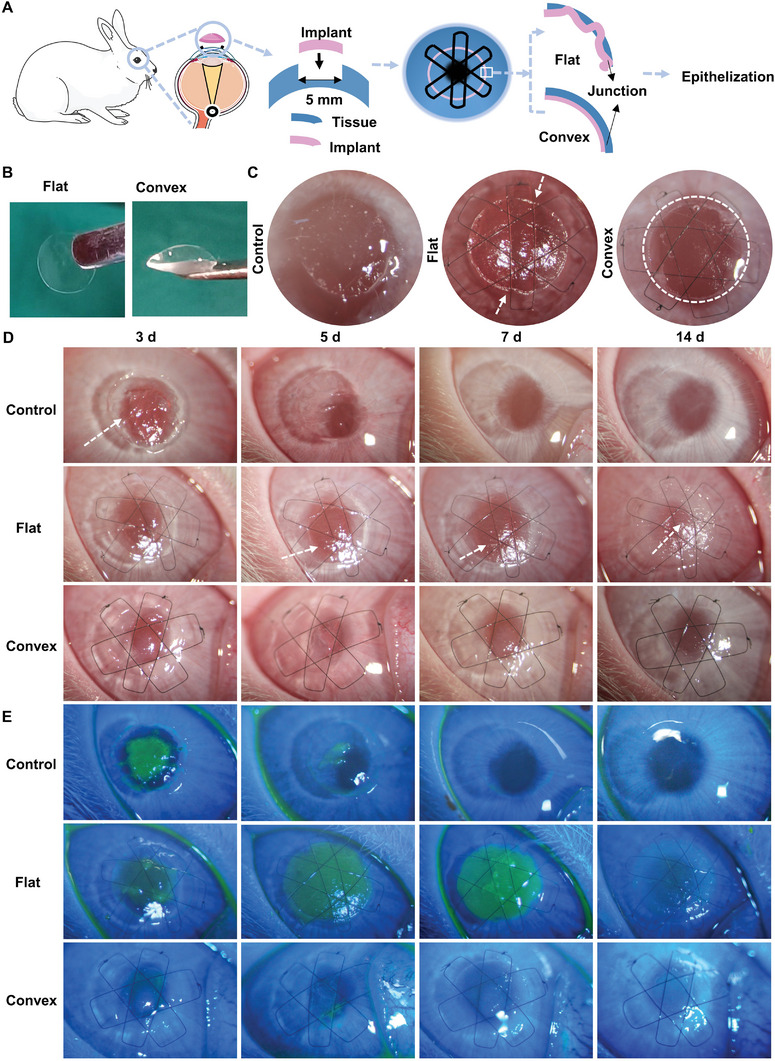
In vivo evaluation of the 3D‐printed cornea in a rabbit model of lamellar keratoplasty. A) Schematic of the superiority of convex implants. B) Images of printed flat/convex implants before being implanted into rabbit models. C) Images of cornea implants taken by slit lamp after operation in a rabbit keratectomy model, the white lines indicate warped implants. D,E) Images taken by slit lamp at 3, 5, 7, and 14 days of transplantation of the 3D‐printed flat/convex cornea, with no material as control, and the white lines indicate the bare implant bed. Bright‐field images (D) and sodium fluorescein staining (E) images showing the eye condition and reconstruction efficiency of the three groups.

To further investigate whether material topography (flat/convex) can affect corneal repair, 3D‐printed flat and convex G10C0.6 implants were used in a rabbit corneal lamellar transplant as experimental groups, as shown in **Figure** **5**A, while only corneal defects were created without any materials on the wounded recipient bed as a control. It was obvious that flat implants warped on the junction with the recipient bed, while the convex group fit in well with all the defective parts after the operation, which could be attributed to the greater plasticity of the curved structure (Figure 5B,C). From gross observation of Figure 5D,E, obvious collagen fibers of the recipient bed were seen before the epithelialization process was completed in the control group, the implanted convex cornea was robust enough without any cracks and in situ throughout the observation, the material remained transparent, and no obvious inflammation or neovascularization was seen. Flat implants deteriorated at the margin by day 3 with rheum, and the damaged parts developed with time; notably, the implants melted completely by day 7. Further regeneration of the flat group was similar to that of the control group because the recipient bed epithelialized on day 14. It was obvious that epithelial cells reached confluence in the convex group on day 3, while only a small part around the defect was epithelialized in the flat group. The epithelialization procedure remained stable for convex implants during the entire experiment, whereas the flat group failed to complete regeneration owing to the melting of implants. In the control group, epithelialization of the margin was observed on day 3, and it was completed on day 7. After 14 days of observation in the three groups, the regeneration advantages of convex implants were prominent, especially when the flat implants melted gradually.

### 2.6 Epithelium and Stroma Restoration Can be Enhanced by Printed Implants

**Figure 6:**
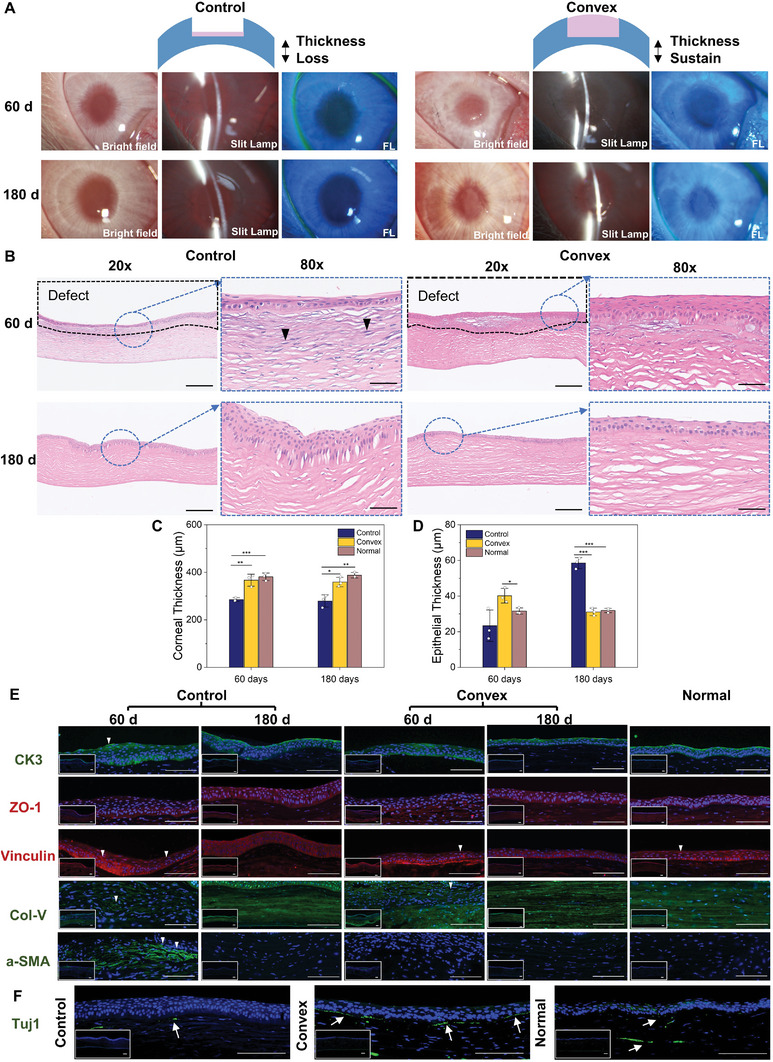
Postoperative evaluation at days 60 and 180 in a rabbit model of lamellar keratoplasty. A) Images of cornea implants taken by slit lamp after operation at days 60 and 180 in a rabbit keratectomy model (FL means fluorescein staining). B) Typical hematoxylin and eosin (H&E) staining in the control wound group, convex group, and normal group on days 60 and 180 postsurgery. The black marked parts are defects and the blue parts are magnified. The scale bars of the upper images are 200 µm, and the scale bars of the magnified images are 50 µm. C,D) Thickness of the total and the epithelium layer of cornea at 60/180 days postsurgery obtained from the H&E image. E) Immunofluorescent staining in all groups at days 60 and 180 postsurgery including CK3 (green), Col‐V (green), ZO‐1 (red), vinculin (red), and *α*‐SMA (green) antibodies. The white triangle indicates typical differences. In all images, nuclei were costained with DAPI (blue). F) Immunofluorescent staining of corneal neurons (green) in all groups at day 180. The white arrows indicate typical neurons, Tuj 1 indicates beta‐III tubulin. The scale bars in (E,F) are 100 µm.

For long‐term regeneration, 3D‐printed convex cornea was selected as the experimental group, and its biocompatibility for regeneration of corneal defects was evaluated to demonstrate the regenerative and functional capacity of the biomimetic printed cornea on damaged wounds. At both day 60 and day 180 posttransplantation (**Figure** **6**A), all groups maintained stable epithelialization and smooth surfaces, and slight nebula appeared in the convex group at day 180, which was speculated to vanish with time. At the early stage of regeneration (day 60), similar to the normal group, the regenerative stroma exhibited ordered collagen fibers of the convex group, only few inflammatory cells were observed, and the epithelium exhibited the three typical layers and similar thickness as the native cornea. However, hematoxylin and eosin (H&E) staining of the control group showed that large amounts of inflammatory cells had infiltrated the stroma, and there was an apparent decrease in thickness, which is considered to be a pivotal indicator for vision (Figure 6B). On day 180, the control group still lacked fundamental thickness, accompanied by epithelial hyperplasia. For the convex group, it can clearly be seen that most of the recovered and regenerated collagen fibers were tightly and well aligned (Figure 6C,D), which indicates that the implants can act as an efficient substitute for promoting tissue regeneration.

Cytokeratin 3 (CK3) staining^[^ ^19a^ ^]^ showed an epithelial layer and a mature type of cornea (Figure 6E), and epithelium migrated over the stroma in all groups, indicating cell maturation and stability as the epithelialization progressed. As epithelial cell adhesion is important for corneal regeneration and we found that convex implants could promote cell adhesion, we investigated typical proteins related to epithelial adhesion. Zonula occludens‐1 (ZO‐1) protein, which is involved in tight junctions between cells, showed that the convex group presented tight junctions through all layers similar to the native cornea, while ZO‐1 protein staining was observed only in the basal layer of the epithelium of control groups at day 60. In the normal and convex groups, vinculin expression in epithelial cells was high around the cell membrane, but some cells lacked or overexpressed vinculin expression, resulting in abnormal epithelial functions in the control groups at day 60, which played a fundamental role in promoting cell adhesion. Collagen V is a quantitatively minor regulatory fibril‐forming collagen responsible for the formation of heterotypic fibrils^[^ ^38^ ^]^ and can reflect stromal regeneration. Collagen V^[^ ^38^ ^]^ secreted by keratocytes showed that the regenerated fibers of the control group were fewer and more disordered than those of the convex group and normal cornea, with dense collagen fibers growing with the degradation of materials, while materials acted as an implant to induce organized regeneration. *α*‐Smooth muscle actin (*α*‐SMA) is a typical protein that is associated with abnormal ECM and scar formation. A large amount of *α*‐SMA was expressed in the defective area of the control groups at day 60, while the convex group and normal cornea did not express much, indicating that regeneration with printed implants was conducive to restraining fibroblast formation. More importantly, corneal neuron fibers appeared to be regenerated in the convex group at day 180 (Figure 6F), indicating that visual functions were recovered. Taken together, printed convex implants can be considered to be good cornea substitutes in terms of both structural and functional aspects.

## 3 Discussion

The cornea is an anisotropic structure with curvature, and its reproduction using traditional casting methods is challenging because of the balance of sophisticated topography, transparency, mechanical strength, and biocompatibility. Moreover, anisotropy results in different regions of the cornea having distinct stiffness and adhesion properties.^[^ ^10^ ^]^ Curvature, as a typical topography in vivo, plays a pivotal role in regulating cell growth on cell‐scale surfaces, but whether milliscale surfaces can affect cell behavior or the mechanism is still unknown. Previous research has used 3D printing to generate curvature or aligned fibers to stimulate the native corneal structure. However, owing to the limitations of inks and printing conditions, step effect induced by layer‐by‐layer or point‐to‐point modes can readily occur. Although some efforts have been made to fabricate completely curved structures,^[^ ^5^, ^8^, ^39^ ^]^ the surface was not smooth, and these studies emphasized mostly in vitro applications. Based on their endeavors to print the cornea, we selected GelMA as one of the components to achieve successful extrusion and deposition, with collagen as the other component to increase printability and implant properties. In order to obtain smooth corneal implants, it was crucial that inks were somewhat liquid‐like to spread on the platform but that they could also remain constant with the help of temperature without redundant sprawl. Based on this principle, we found that an extrusion temperature of 27.5 °C and a platform temperature of 20 °C were the most optimal printing parameters to obtain corneal implants with a smooth and controllable thickness of at least 186 µm. Interestingly, the structure of the printed cornea under the optimized condition exhibited continuous thickness variation, with a thinner center and thicker periphery, which was nearly the same as that of the native cornea and rare in the studies to date. This was attributed to the fact that inks should spread slightly without sprawl, and some inks spread outwards under the effect of gravity.

During the process of culturing RCECs, it was surprising that cells on convex structures grew slower than those on flat structures, and they tended to align as a circle, so we decided to further investigate the mechanism of how slope gradient on convex curvature mediated cell behavior. Cells position themselves in complex three‐dimensional (3D) environments that exhibit multifarious topographical features in vivo. Their proliferation and functions are tightly connected to cellular interactions with extracellular matrix (ECM), and increasing evidence has recently shown that environmental signaling to cells entails much more than biochemical cues.^[^ ^12^ ^]^ Physical cues such as stiffness, roughness,^[^ ^40^ ^]^ and topography^[^ ^22^ ^]^ have been recognized as decisive factors that mediate cell behavior. Curvature, which is one of the most common topographies in vivo, can affect cell fate during development and is closely associated with diseases such as cancer.^[^ ^13^ ^]^ Pioneering work using ultrasmooth 3D sinusoidal topographies has shown that cells tend to position themselves in concave valleys through nucleus and cytoskeleton actuation, which eventually affects FA organization and dynamics.^[^ ^14a^ ^]^ Despite the efforts made to understand the impact on cell behavior, the substrates used were mostly resins that were suitable for structural research but did not exhibit excellent biocompatibility. In addition, these studies concentrated on nano‐ or micronscale surfaces, and the tissue‐size (milliscale) curvature was largely overlooked and underestimated.^[^ ^14^, ^40^, ^41^ ^]^ The obstacles were as follows: first, the ECM‐like curvature substrates were difficult to produce, as mentioned above; second, inoculating cells on curved surfaces was challenging owing to the 3D structure. As epithelial adhesion determines whether corneal epithelial wounds heal well and cells often react to microstructure, we investigated the mechanism of curvature‐mediated cell behavior and adhesion and illustrated the relationship between the mechanism and corneal regeneration. In the present study, we observed that not only can the milliscale curvature guide cell alignment, but different slope gradients on it can also mediate cell behavior and adhesion, which provided new insight for designing material topography.

When RCECs were cultured on surfaces with different slope gradients, the cell alignment was in line with the slope gradient increase, and they preferred to grow along the longitudinal axis, with slow growth along the latitudinal axis because the cells tended to avoid inclination, which is a topography that requires more geopotential energy. Cells can also sense topography variations and regulate their cytoskeleton and nuclei to maintain the total potential energy minimum.^[^ ^42^ ^]^ When cells were on steeper slopes, their cytoskeletal shape became more fusiform, with a smaller volume, and the nuclei were compressed, resulting in more chromatin condensation. From the protein semiquantification analysis, it was not surprising that cells positioned on a steeper slope exhibited better adhesion. Additionally, we described a new mode of RCEC organization in response to physical cues mediating cell alignment and adhesion, and we explained this finding through FEM simulation. Cell layers were regarded as elastic springs, and in Part IV, the shear stress was larger, so cells exhibited alignment and morphological changes. Additionally, the expression of adhesion‐related genes and proteins were increased, so the density of the adhesive molecules increased. Through FEM analysis, we could simulate the traction force further, which verified the increase in adhesion.

**Figure 7:**
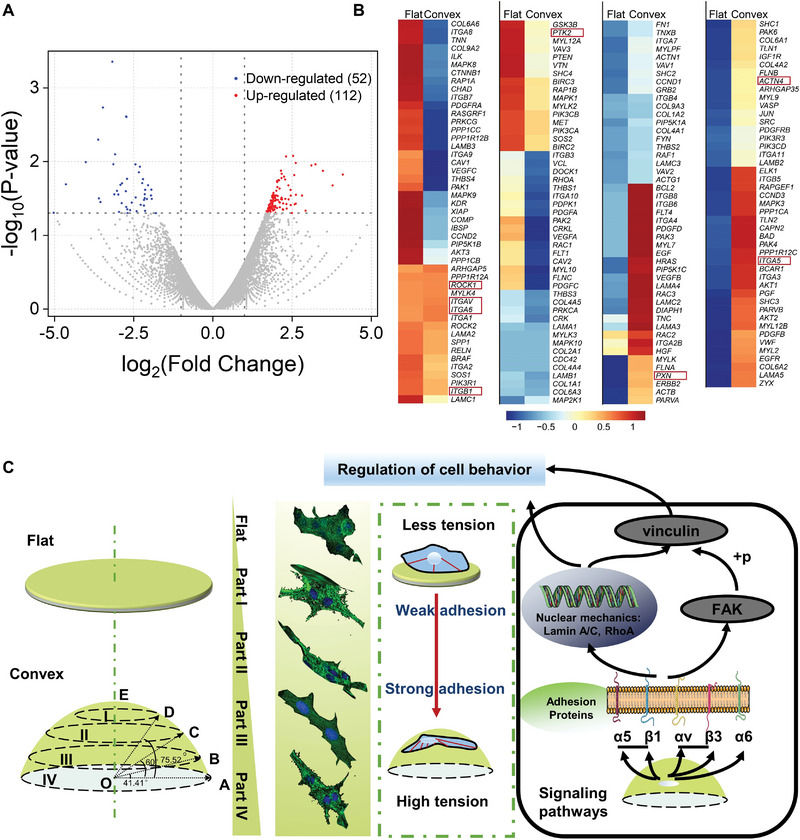
Schematic representation of the proposed slope gradient‐induced changes in rabbit corneal epithelial cell (RCEC) behavior and signal transduction pathway prediction. A) Volcano plot of all differentially expressed genes of RCECs when seeded on flat or convex corneas, with the former as control. B) Specific gene expression of focal adhesion (FA) pathway components for flat and convex corneas. Red and blue blocks indicate upregulation and downregulation of gene expression compared to the flat group, and the genes in red squares are typical genes in the integrin‐based pathway. C) When cells were located on a steeper slope, cytoskeletal forces stimulated a large push force toward the surface, resulting in compression and deformation of the nucleus. The higher tension on the nucleus induced higher *RhoA/LmnA 7* expression levels and promoted adhesion. Owing to the limited vertical extension of the cell, only a small push force was imposed on the nucleus on flat surfaces. Slope gradient activated integrin through adhesion protein absorption, and FA proteins were then activated. These signals influenced most of the adhesion behaviors.

Some studies have shown that cell adhesion is initiated by the activation of *α* and *β* integrins of the cytomembrane, which in turn are activated by biochemical and topographic properties of ECM surfaces.^[^ ^43^ ^]^ We then studied whether convex topography, in addition to changes in cytoskeleton shape, intracellular tension, and adhesion properties, could affect gene expression in RCECs. We compared the transcriptome of RCECs on printed flat versus convex corneas, which revealed 52 highly expressed genes (red dots in **Figure** **7**A) and 112 downregulated genes (blue dots in Figure 7A) compared to the flat group. From the identification of 186 genes in the FA pathway (Figure 7B), some genes related to the integrin‐based pathway influencing adhesion and cytoskeleton changes, including the integrin family (*ITGAv*, *ITGA5*, and *ITGA6*), FA genes (*PXN* and *PTK2*/*FAK*), and others related to the integrin‐based pathway (*ACTN1*, *ROCK1*, etc.). In addition, the results of RNA‐seq and qPCR together illustrate the pathway in this study, as shown in Figure 7C, where the interfacial slope gradient cues are transduced into biochemical factors via the integrin‐based adhesion and transduction signaling pathway. Integrins *α*5*β*1, *α*v*β*3, and *α*6 directly connect and transmit external traction from substrates, facilitating FA formation by connecting extracellular substrates to F‐actin fibers extending from within the cell, for which integrin *α*v*β*3 is more representative in this static model. The adhesion was stable only when sufficient force was exerted upon adhesion to recruit integrins, whereas the signal on the flat substrate was weak. Integrin clustering then initiated the pFAK‐vinculin and RhoA‐lamin A/C pathways to transduce the slope variation stimulation to mediate cell behaviors and influence cell adhesion. Understanding these internal mechanisms can guide material topography design and clarify their roles in the regulation of curvature sensing.

Through material design and mechanistic research, we created a printed convex cornea similar to the native cornea, while suppressing step effect, which can also promote cell adhesion owing to its unique topography. We further verified its superiority through rabbit lamellar keratoplasty, and the results showed that it can fit well with neighboring tissue, exhibit stronger epithelium adhesion, and induce more collagen and neuron fiber regeneration. More specifically, the 3D‐printed convex cornea maintained normal epithelium types and functions based on staining results of CK3, ZO‐1, Col‐V, and vinculin proteins. Moreover, it can promote stromal regeneration with the degradation of materials. Compared with existing printed corneas or other materials, the efficiency was obvious when the defect was serious, while some reported defects were less than 3.5 mm in diameter.^[^ ^19a^ ^]^ Our data suggest that printed convex implants are novel versatile substrates for culturing corneal epithelium and stimulating cell adhesion, and they are suitable for corneal transplantation by promoting epithelization and stroma regeneration. Our concept correlated curvature topography with function execution to create a versatile corneal implant that can be applied in the field of corneal damage. This can guide material design and provide a new strategy for corneal regeneration. Altogether, this study established slope gradient on curvature as a new cellular guiding mechanism. We propose that by affecting gene/protein expression and cell adhesion, milliscale curvature and its slope gradient variation can be considered vital regulatory cues. In vivo experiments also verified the potential clinical applications and accelerated the progress in the field of 3D printing.

## 4 Experimental Section

### 4.1 Printing Procedure and Optimization

A pneumatic, dual extruder 3D printer was used to generate the cornea implants on the basis of a preset pathway. Before printing cornea, traditional grid structures with an area of 7.5 × 7.5 mm, layer thickness, and the distance between two stands of 0.25 and 1.5 mm, respectively, were printed firstly to assess the printability of temperature‐controlled inks under extruding temperatures of 22.5 °C, 25 °C, 27.5 °C, and 30 °C. More detailed printing parameters are listed in Table S1 (Supporting Information), considering that the pneumatic pressure under different conditions can vary according to the nozzle temperature. The printability of the inks was calculated as the printing value (Pr), which was determined by the grid perimeter (*L*) and area (*A*) as Pr = *L* ^2^/(16*A*). In a typical corneal experiment, the printing speed of the nozzle in the *x*–*y* direction was set at 6 mm s^−1^, and 0.26 mm (inner diameter) high‐precision blunt needles (Regenovo, Hangzhou, China) were used in all printing experiments. The printing gap was set as 0.4 mm, and the thickness of the printed structures was determined mostly by the nozzle speed and pneumatic pressure. G10C0.6 inks were chosen to investigate the optimal conditions for printing corneal implants. The GelMA/collagen inks were loaded in a 5 mL syringe, and the temperature of the nozzle was set before calibrating the apparatus. In this step, GelMA/collagen inks underwent a rapid liquid‐gel transformation under different nozzle temperature controls, and there was a big difference when the inks were squeezed out from the needle. The temperature of the high‐throughput corneal platform was maintained at 20 °C to keep the printed hydrogel equitable. Subsequently, the deposited corneal structures on the platform were cross‐linked under a UV light source (200 mW cm^−2^) at a UV projector speed of 8 mm s^−1^, which was sustained for 45 s to ensure the stability of the printed cornea implants. 3D‐printed flat structures were also printed as controls in the following experiments. The printing method for constructing 3D‐printed flat structures was similar to that for convex structures, with the difference that flat structures were printed on a flat substrate from outwards to inward forming a circle, while convex structures were printed from the bottom upward. The GelMA/collagen inks used in this study were 10% (w/v) to 0.6% (w/v), as shown in Figures S3 and S7 (Supporting Information).

### Physiochemical Characterization

Images of grids taken with a stereo microscope (Stereo Discovery.V20, Zeiss, Germany) were selected to calculate the Pr and study the temperature variation of the G10C0.6 inks, and Rhodamine B (Sigma–Aldrich, ≥ 95%) was applied to immerse the implants for visualization of the grid shape. The surface morphology of the 3D‐printed cornea implants was investigated using an ESEM (Novo Nanosem 430, USA) at an accelerating voltage of 20.0 kV in a low vacuum to maintain the original shape. OCT sectioning (Spectralis HRA + OCT, Heidelberg, Germany) was used to visualize the entire structure and thickness change of 3D‐printed cornea implants under different temperatures with implants laid on a convex mold to preserve their original shapes, and the thicknesses at both the center and the periphery were measured using ImageJ software. The internal cavity micromorphology structures were observed using a scanning electron microscope (SEM, Merlin, Germany) at a voltage of 20 kV, and the samples were frozen in liquid nitrogen and quickly fractured before being treated by spraying gold.

### Cell Trajectory

RCECs were maintained in DMEM (Gibco) supplemented with 10% fetal bovine serum (FBS, Gibco) before use. 3D‐printed cornea implants were sterilized by immersion in 75% alcohol overnight and placed in PBS solution for 1 h to wash off the remaining alcohol. A high cell density suspension was prepared to track the RCEC pathway on 3D‐printed convex cornea of G10C0.6, which was proven to have comprehensive properties prior to the other components, and the convex structures in the following experiments were G10C0.6. Ten cell suspension droplets were evenly scattered onto the material, each volume of which was 1 µL containing 5 × 10^3^ cells, and another 500 µL of medium was then added into the well 2 h later to continue the culture. On days 1, 3, 5, and 7, an inverted microscope (Olympus, Japan) was used to screen for cell aggregates, as shown in Figure S10 (Supporting Information). Furthermore, the size change of the cell cluster in the longitudinal and latitudinal directions was analyzed using ImageJ, and the size of the cell cluster in the longitudinal direction was recorded as length, while the latitudinal direction as width, Δ was calculated by length or width change between the chosen days of 3, 5, 7, versus 1. More specific details were imaged using an inverted fluorescence microscope (Axio Observer.7, Zeiss) with bright‐field imaging when the cells were inoculated for 7 days.

### Adhesion Force

RCECs inoculated on different slope gradient positions at 3D‐printed convex cornea structures and 3D‐printed flat structures were chosen to study the effect of slope gradient on adhesion force. Cell suspension droplets (1 µL) with 2 × 10^3^ cells were slowly applied at the specified location of Parts I–IV and flat implants with the same surface area. The culture procedures were the same as those mentioned above. The adhesion force was quantitatively determined using a centrifugation assay according to the method proposed by Reyes and Garcia.^[^ ^27^ ^]^ Briefly, samples were rinsed three times with PBS to remove apoptotic cells and medium, followed by incubation in 2 µg mL^−1^ calcein‐AM solution for 15 min, while avoiding light, after being cultured for 5 days to stain all live cells. The original number of cells on the samples was counted under an inverted fluorescence microscope after rinsing with PBS, and the centrifugation assay was performed when samples were placed at the bottom of centrifuge tubes with the side culturing cells downwards in PBS and centrifuged at 300 and 1000 rpm for 5 min. The remaining cells on the samples were counted using an inverted fluorescence microscope according to a previously reported method.^[^ ^44^ ^]^

### Immunofluorescence

RCECs were stained for characterization after 5 days of culture, as described in the procedure for adhesion force measurement. The samples were rinsed three times with PBS, fixed with 4% paraformaldehyde (Beyotime Biotechnology, China) for 30 min at room temperature, permeabilized in 0.1% Triton‐X 100 (Beyotime Biotechnology) for 15 min, and then incubated with 1% (w/v) bovine serum albumin (BioFroxx, China) for 30 min to block nonspecific binding. The samples were incubated with primary antibodies against vinculin (Proteintech, USAdilution, :1:400) overnight at 4 °C, washed three times with PBS, and then incubated with goat anti‐rabbit (Alexa Fluor 594, Abcam, dilution:1:200) secondary antibody for 1 h at room temperature in the dark. Alexa Fluor 488 phalloidin (1:200, Beyotime Biotechnology) was added for 1 h to stain F‐actin. Finally, the cells were stained with DAPI (1:200, Beyotime Biotechnology) for 15 min before observation using a Leica TCS SP8 microscope. To allow comparison of the fluorescence intensity of different sample, the exposure time and the laser power were kept constant, and the method to acquire image stacks was the same to eliminate external errors.

### Data Analysis of Immunofluorescence

*Z*‐stacks were recorded at each step of 1 µm to obtain 3D reconstructed images of cell morphology, and images were analyzed using ImageJ software to ensure consistent analysis. For the vinculin fluorescence intensity, all laser scanning confocal microscope settings were kept the same and the image acquisition was performed using a 63× oil‐immersion objective with optimal *Z*‐sectioning parameters. The average signal intensity per cell was calculated by multiplying the pixel intensity values by the number of pixels that contained this intensity, and these were then divided by the total cell number. All intensity data were obtained using Leica TCS SP8 analysis software. For nuclear quantification, the DAPI fluorescence intensity was used to measure chromatin condensation, and nuclear volumes were reconstructed from *Z*‐stacks of DAPI‐stained cells using ImageJ software (all steps were performed at room temperature unless stated otherwise).

### Gene Expression Analysis

RCECs cultured on flat or convex structures were collected for gene expression analysis. The culture procedures were similar to those for cell proliferation, except for the cell density of 2 × 10^4^ cells µL^−1^. After inoculation for 5 days, RCECs were collected and lysed using TRIzol reagent (Invitrogen) to extract RNA using a HiPure Total RNA Micro Kit (Magen). The RNA quality and concentration were determined using a NanoDrop 2000 spectrophotometer (Thermo Scientific, USA). A PrimeScript RT reagent kit with gDNA Eraser (TaKaRa Biotechnology, Japan) was used to reverse transcribe the same amount of RNA into cDNA according to the manufacturer's protocol. The primers for the target gene are listed in Table S2 (Supporting Information). Real‐time PCR detection was performed using SYBR Green reagents (GeneCopoeia) on an RT‐PCR instrument (QuantStudio 6 Flex, Life Technologies) with 40 amplification cycles. Cells cultured on flat structures were used as a reference for comparison. GAPDH was used as an endogenous control for normalization of the expression levels of genes using the ΔΔCt method.^[^ ^45^ ^]^

### Western Blot Analysis

Western blotting was performed according to standard procedures. Briefly, cells were lysed in radioimmunoprecipitation assay (RIPA) buffer, and total protein was estimated using a colorimetric Micro BCA Protein Assay (Thermo Scientific). Fifteen micrograms of total protein sample were loaded on precast Mini‐PROTEAN TGX Stain‐Free gels and separated by electrophoresis. A ChemiDoc imaging system was used to normalize the protein gel images before transfer using a Trans‐Blot Turbo system. The membranes were then blocked with 3% BSA (Sigma–Aldrich, France) for 1 h at RT. Primary antibody incubation was performed overnight at 4 °C using the following antibody concentration: vinculin (1:2000, 26520‐1‐AP, Proteintech). After short rinses, the blots were incubated with secondary antibody (1:3000) for 1 h at RT. Protein bands were detected using a ChemiDoc imaging system. Relative protein levels were quantified using ImageJ software by normalizing the levels of target proteins to GAPDH.

### Rabbit Lamellar Keratoplasty and Evaluation

All animal experimental procedures in this study were approved by the Animal Ethics Committee of the South China University of Technology (AEC No. CV2022003). 3D‐printed flat/convex corneas with composites of G10C0.6 were selected for anterior lamellar keratectomy, which was performed on male New Zealand albino rabbits (weight: 1.8–2.0 kg) under anesthesia by intramuscular administration of 2% pentobarbital sodium (30 mg kg^−1^). The right eye underwent anterior lamellar keratectomy with materials as the experimental group, while the wounded bed without materials was used as a control. A 5 mm corneal trephine (Shiqiang Co., Ltd., Suzhou, China) and crescent knife (Sharpoint, USA) were used to create approximately 200 µm of anterior corneal tissue in the center of the cornea, and the tissue was removed by manual lamellar dissection with a crescent knife. 3D‐printed flat/convex corneas with thicknesses of 200/185 µm and a diameter of 5 mm were then implanted by overlying sutures^[^ ^46^ ^]^ using 10‐0 nylon sutures (Alcon, USA), which were anchored just in the host tissue and laid on the top of the implant surface, keeping it in place. After surgery, 0.3% tobramycin (Alcon Laboratories) eye drops were applied to the surgical eyes three times a day. Gross observations, ophthalmic microscopy (slit lamp, Topcon system), and fluorescein staining were performed 3, 5, 7, 14, 60, and 180 days after surgery to examine material transparency, curvature, neovascularization, and re‐epithelialization. The sutures were removed on day 30. At 2 months and 6 months after surgery, the rabbits were sacrificed and the implanted site was excised and fixed in 4% paraformaldehyde to prepare paraffin sections for H&E staining. Paraffin sections were viewed under a light microscope (Axio Imager Z2, Zeiss). For immunostaining, paraffin sections were fixed and subjected to immunofluorescence staining. Every 10 µm thickness was sectioned for staining using procedures similar to those described above, except that the primary antibodies were against CK3 (sc‐80000, Santa Cruz Biotechnology), ZO‐1 (bs‐23835R, Bioss), vinculin (26520‐1‐AP, Proteintech), Collagen V (bs‐0552R, Bioss), *α*‐SMA (F3777, Sigma–Aldrich), and Tuj1 (AT809, Beyotime).

### RNA‐Seq Analysis

RNA was isolated as described in the Gene Expression Analysis section. After RNA extraction and purification, library construction and sequencing were performed at Personalbio Corporation (Shanghai, China) to conduct next‐generation sequencing based on the Illumina sequencing platform (USA). To further assess the integrin‐based pathway, gene set enrichment analysis (GSEA) was used to analyze the differentially expressed genes of the FA pathway.

### Statistical Analysis

The data presented herein are expressed as mean ± standard deviation (SD) unless stated otherwise. Each experiment was repeated at least three times using biologically independent samples. Statistical analysis was performed using one‐way ANOVA with multiple comparison analysis using Tukey's post hoc test, and *p*‐values < 0.05 were considered statistically significant (*p < 0.05; **p < 0.01; ***p < 0.001). All statistical tests and graphs were generated using Origin 9.0 software.

## Conflict of Interest

The authors declare no conflict of interest.

## Author Contributions

Y.X., J.Y., B.J., W.S., and R.L. supervised the study. J.L. performed the animal experiments under the supervision of X.S, Q.Z., H.L., and Y.P. Q.W. conducted the FEM simulation experiments. Y.H. designed the illustrations and edited the manuscript. All the authors contributed to the discussion and writing of the manuscript.

## Supporting information

Supporting Information

Supplemental Movie 1

Supplemental Movie 2

Supplemental Movie 3

Supplemental Movie 4

## Data Availability

The data that support the findings of this study are available from the corresponding author upon reasonable request.
