# Supplementary material for: Biomimetic Convex Implant for Corneal Regeneration Through 3D Printing
Source: Adv Sci (Weinh). 2023 Feb 12;10(11):2205878. doi: 10.1002/advs.202205878 (PMC10104657; doi:10.1002/advs.202205878)
Supplement: Supplementary file 1 — Supporting Information [file ADVS-10-2205878-s001.pdf]

ADVANCED SCIENCE
Open Access

Supporting Information

for *Adv. Sci.*, DOI 10.1002/advs.202205878

Biomimetic Convex Implant for Corneal Regeneration Through 3D Printing

Yingni Xu, Jia Liu, Wenjing Song, Qianchun Wang, Xiaomin Sun, Qi Zhao, Yongrui Huang, Haochen Li, Yuehai Peng, Jin Yuan*, Baohua Ji* and Li Ren**

Supporting Information for:

Biomimetic convex implant for corneal regeneration through 3D printing

*Yingni Xu, Jia Liu, Wenjing Song**, *Qianchun Wang, Xiaomin Sun, Qi Zhao, Yongrui Huang, Haochen Li, Yuehai Peng, Jin Yuan**, *Baohua Ji**, *Li Ren**

Supporting Figures:

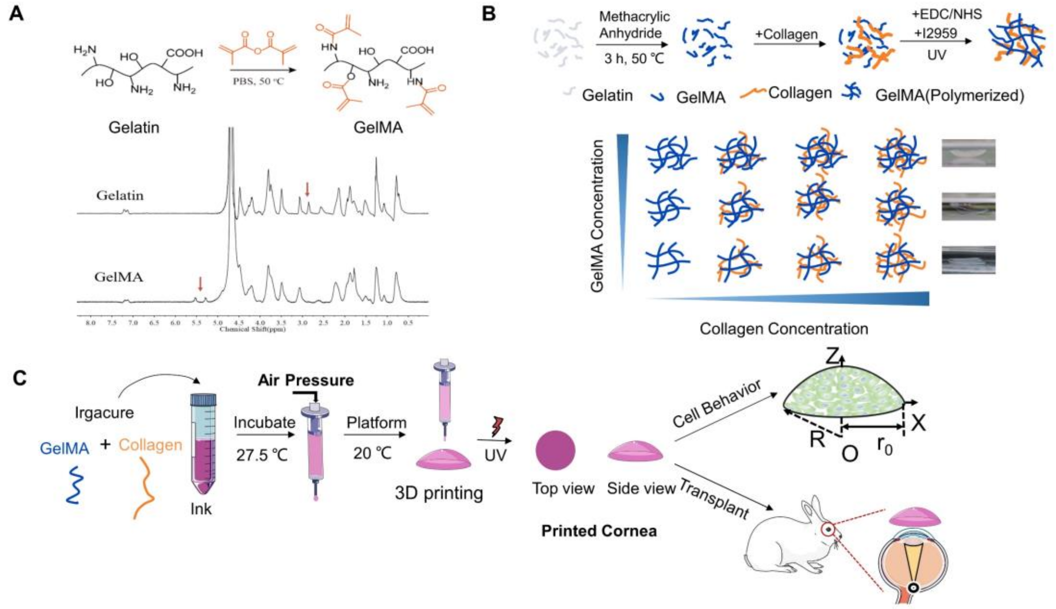

Figure S1. The schematic illustration of the experimental process. A) Characterization of GelMA (grafting ratio of 98%). B) An interpenetrating network of GelMA and collagen to allow for independent tuning of implant stiffness. C) 3D printed cornea directed cell behavior and its application.

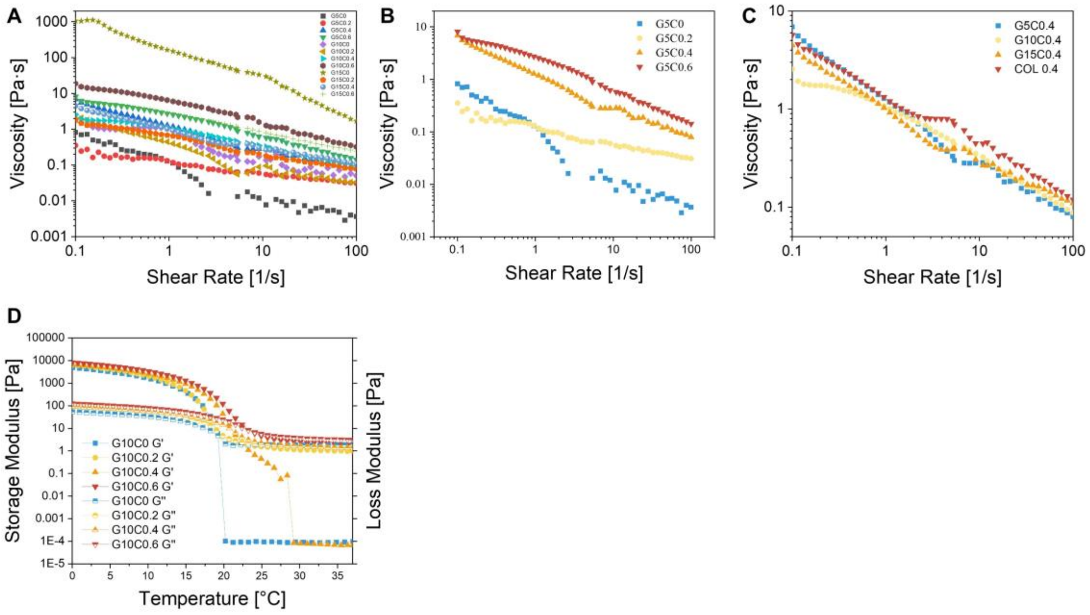

Figure S2. Evaluation of bioinks' printability. A) "Shear thinning" properties of sundry bioinks with different composition of GelMA and collagen. B) Effect of collagen concentration to bioinks' viscosity at 25 ℃. C) Effect of GelMA concentration to bioinks' viscosity at 25 ℃. D) Effect of temperature on storage modulus (G') and loss modulus (G'').

Table S1 Printing Parameters

parameter	value
GelMA concentration（w/v%）	5-15
Collagen concentration（w/v%）	0-0.6
Extrusion pressure（MPa）	0-0.3
Syringe temperature（℃）	10-28
Cooling receiving platform temperatur（℃）	20
XY plotting speed（mm/s）	6-12
UV light intensity（mW/cm^2）	200
UV exposure time after printing of implants (s)	45
Nozzle inner diameter（mm）	0.26

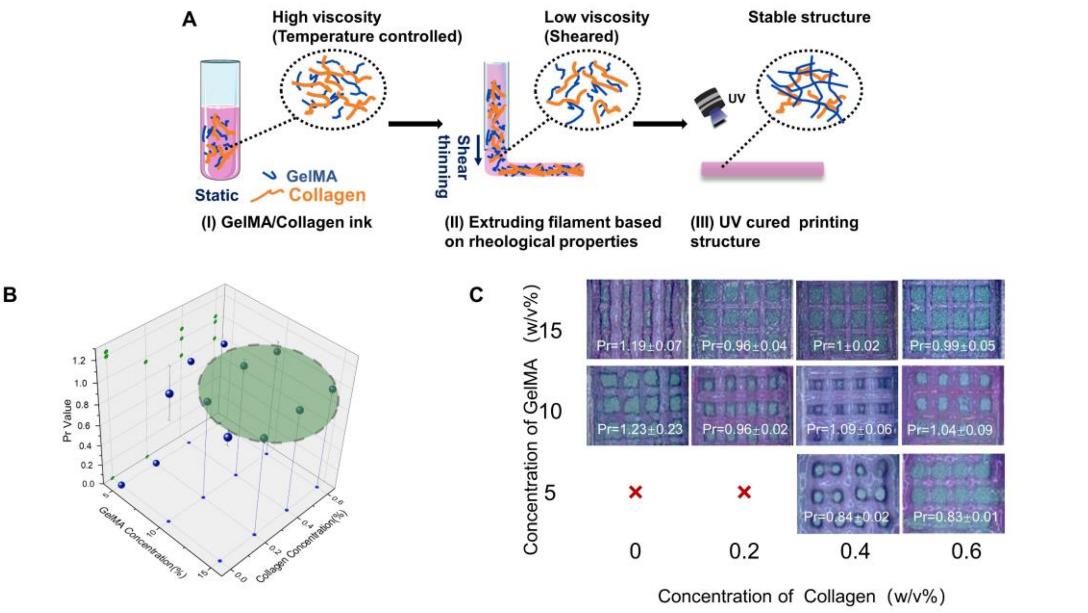

Figure S3. Phase diagram of GelMA/Collagen bioinks' printability under the condition presented in SI Table 1. A) Schematic illustration of printing implants with GelMA/Collagen ink: (I) GelMA/Collagen ink, (II) Extruding filament based on rheological properties, (III) UV cured printing structure. B) Phase diagram of GelMA/Collagen inks' printability (the ratio of GelMA and Collagen marked in dark green circle had excellent printing value (Pr) with regard to the stacking process under different temperature, whose 0.9<Pr<1.1). C) was bright field images of (B).

Printability of GelMA/collagen Inks

As shown in Figure S1, a denser penetrating network was generated with the concentration of GelMA and collagen increased. To confirm that the ink could meet the requirements of extrusion-based 3D printing, the rheological properties of GelMA/collagen inks were measured, namely, shear-thinning (Figure S2A) and rapid-gelling (Figure S2D) properties. It can be seen that collagen played a pivotal role in viscosity compared to GelMA, as the viscosity of G5C0.6 was higher than G5C0 (Figure S2B) while the viscosity of G0C0.4 to G15C0.4 (Figure S2C) were nearly the same. When inks were in static equilibrium in the barrel, temperature controlling can result in different viscosity of inks (Figure S3A(I)). During the extruding process, the ink underwent shearing through the nozzle, due to the shear-thinning property of inks, there was a change into the low viscosity state, which ensured the extrusion from the nozzle successfully (Figure S3A(II)). After extrusion, inks were deposited onto the platform to provide a pre-gel situation, whose temperature was also a decision factor for perfect printing of cornea implants, because low temperature would solid the inks too fast while high temperature

could not stable pre-gel inks, both of which would lead to failure, from temperature-modulus curve in Figure S2D, we made the platform temperature of 20 °C. Finally, the filament was UV cured resulting in a stable scaffold (Figure S3A (III)). To research the optimal inks applied for cornea implants, the printing value (Pr) of traditional grid structures were evaluated to verify good shape fidelity of implants[1], shown in Figure S3b. The printing parameters of this procedure were listed in Table S1, and more detailed pictures were added in Figure S3C. Here, we can see that collagen addition would enhance ink printability and GelMA at 10% and 15% concentrations had better printability while all Pr of G5 were below 0.9, also, natural cornea was a soft structure with curvature while implants at GelMA concentration of 15% were rigid as shown in Figure S1B, so the concentration of GelMA was determined as 10% in the whole experiment with the collagen concentration change. All these layer-by-layer additive steps were the basis of printing in this article, which was regarded as inevitably to produce step effect, an obstacle to realizing smooth and uniform surface of 3D samples.

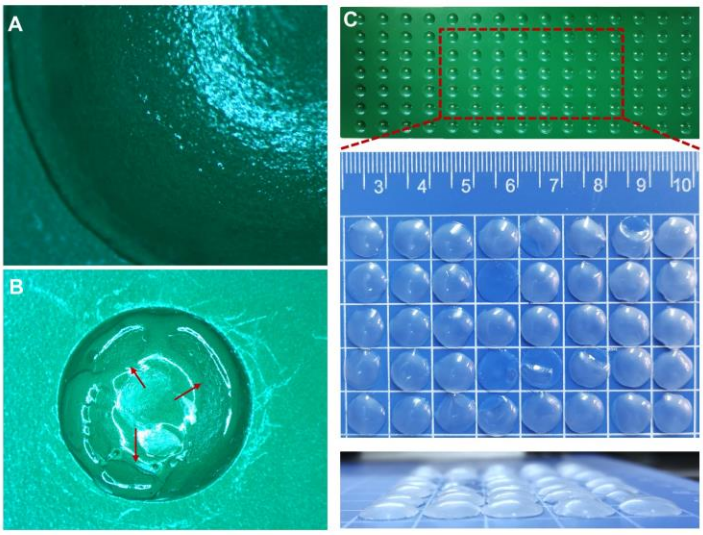

Figure S4. Detailed surface images of 3D printed cornea under different temperature and high-throughput printing cornea images. A) Magnification of pictures in Figure 1(F) at 27.5°C under UV curing. B) Images showed if the thickness was too thin under 30°C, it would fragile resulting failure construct. C) Top view (middle) and main view (lower) images of high-throughput printed cornea implants under the same condition at a time(n=40).

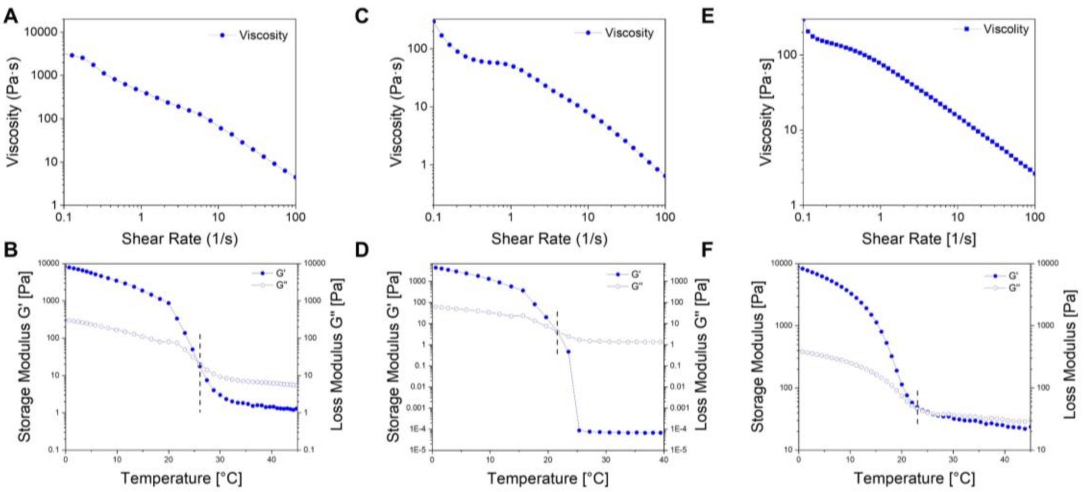

Figure S5. Rheological behavior of other thermal-sensitive inks. "shear-thinning" and "sol-gel" transition properties of Gelatin+Alginate (A-B), GelMA+Laponite (C-D) and GelMA+HAMA (E-F).

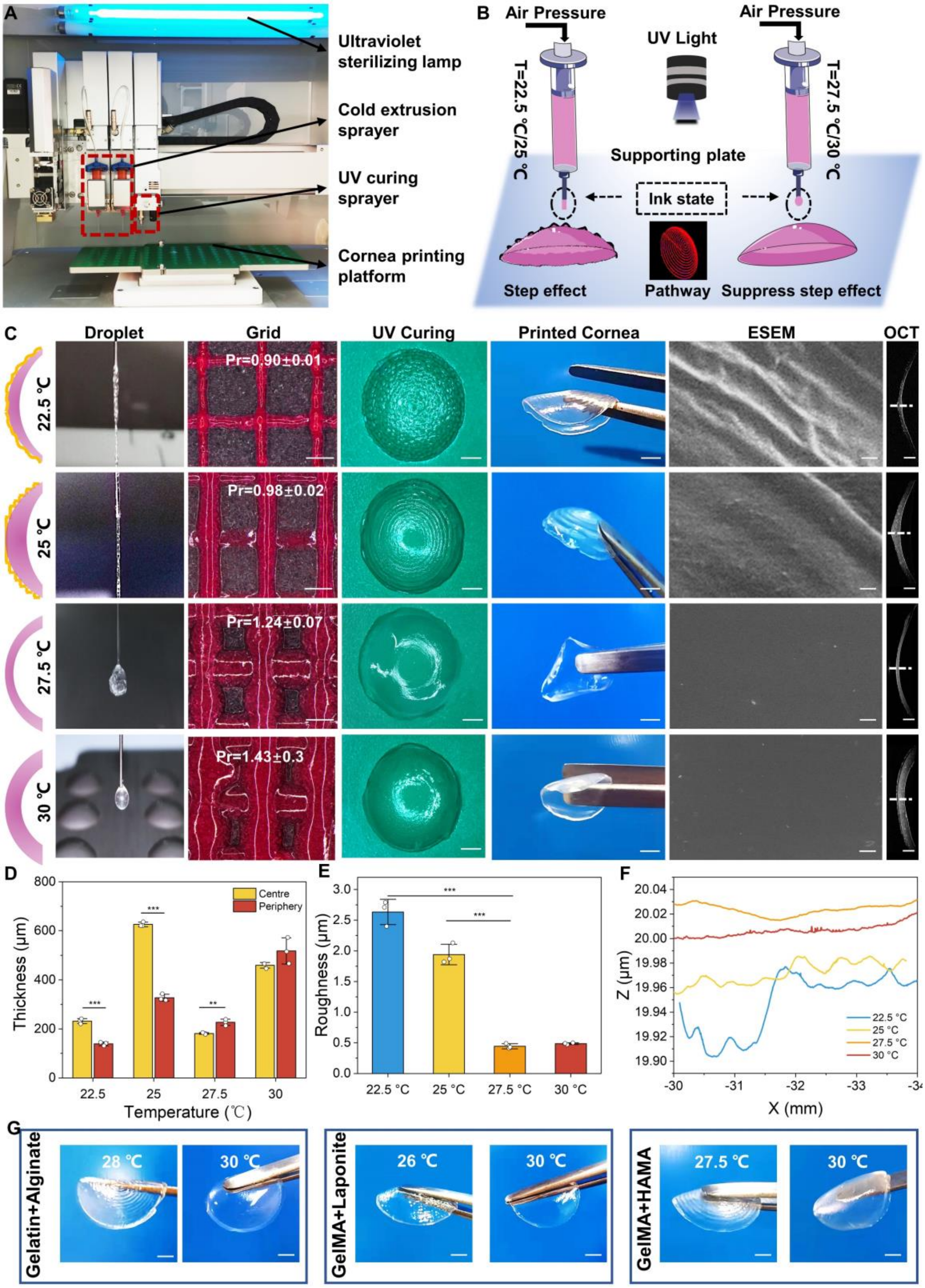

Figure S6. Fabrication of 3D printed convex cornea and strategy of smooth structure construction. A) The custom-made high-throughput 3D printing device of cornea implants. B) Schematic illustration of temperature-controlled printing process to suppress step effect. C) Images of ink deposition at different extruding temperatures and further surface details of printed cornea implants with composite of G10C0.6. Droplet represented the ink situation when it was extruded. Grid showed traditional printing pathway to verify the printability at different temperature, scale bar = 1 mm. Bright field images in UV Curing and Printed cornea displayed actual structure of printed cornea clearly,

while temperature below 27.5 °C had severe step effect, scale bar = 200 μm. ESEM showed its micro surface, scale bar = 200 nm. And OCT exhibited images of printed cornea under different temperature, the scale bar = 1 mm. These all pictures verified temperature plays an important role in surface morphology considering 3D printing cornea. D) Relationship between printing temperature and thickness of corneal structures of G10C0.6. E) Sample roughness at different printing temperature. F) Skeleton diagram of roughness, the change of Z axis showing roughness fluctuation range. G) Universality studies of thermal-sensitive inks based on temperature-controlled 3D printing strategy to suppress step effect, printing temperature (marked in white) were listed in the images. All the results were calculated as mean ± standard deviation (SD) (n=3, biologically independent samples).

Suppression of Step Effect for Printing of Convex Cornea Implants

Inks' properties and printing parameters were the basis of successful construction of corneal implants, and before printing, a range of GelMA/Collagen inks were prepared as shown in Figure S1 (the resulting ink solution were named briefly as GxxCxx according to final concentration). Figure S6A showed the actual high-throughput printing apparatus. As our inks behaved rapid sol-gel transition (Figure S2D), temperature control was the most key factor, another factor was printing pathway as shown in Figure S6B, consecutive pathway ensured no excess ink deposit. As for G10C0.6 ink, the gel temperature was around 25 °C, when extruding temperature was at 22.5 °C, inks tended to form wrinkled filaments at the nozzle outlet and became smooth filaments as temperature increased to 25 °C (Figure S6C), which was the appropriate condition for traditional grid structures printing. At the extruding temperature of 27.5 °C and 30 °C, inks were near at sol state in the extruding procedure, and droplets were created in the meantime. The higher the temperature, the smoother the droplets would be, we can see clearly from grid structures that the filaments were deformed to glabrous lines close to fusion, indicating they can flow to a certain extent. Under traditional printing parameters of 22.5 °C or 25 °C, the printed cornea had serious step effect looking like whorl, which was presented from ESEM pictures. Sample thickness was related to the printing pressure, all pressure presented in Figure S6C were the minimum to create complete cornea implants. When the extruding temperature was 27.5 °C and 30 °C, droplets tended to stream slightly to outwards and downwards along the printing pathway, resulting in the center of implant was thinner and the periphery was thicker, as displayed in Figure 1D, which was nearly the same as natural corneal structure. Owing to the near sol state of deposited inks, casting phenomenon came into being to generate smooth surface as shown in ESEM pictures and Figure S4A. Especially, even temperature of 30 °C can suppress step effect, it can not produce thinner samples as the viscosity

was too low (~570 μm), there was not enough connection between droplets, and hollow structures would appear if printing thin implants, seen in Figure S4B. From OCT sections, all implants had curvature structures with different thickness characteristics, temperature below 25 °C produced implants with a thick tip, and with temperature increased to 25 °C, the tip effect was more apparent up to 625 μm. Temperature at 27.5 °C processed the most similar advantages of natural cornea with average thickness lowest to 186 μm, which was acquired under the minimum air pressure when inks can be extruded, and to gain various thicker cornea, we can increase either air pressure or extruding temperature to fulfill overall corneal personalization. From Figure S6E-F, we can see that sample roughness of 27.5 °C or 30°C were 0.5μm, which can be determined as smooth surface with nearly no surface fluctuation, but sample roughness of other temperatures were around 2 μm with apparent processing traces, conforming to ESEM pictures. Additionally, to verify the temperature-controlled method of suppressing step effect was effective, we then chose another three inks possessing thermal sensitive typically (Figure S5), it's interesting that all they can be applied in this printing system to alleviate step effect (Figure S6G). Moreover, this technique can be applied in high through-put printing, and implants with 8 x 6 numbers can be made in a time (Figure S4C and movie S1) within 50 minutes, which is persuasive to show the stability and efficiency of corneal printing technique.

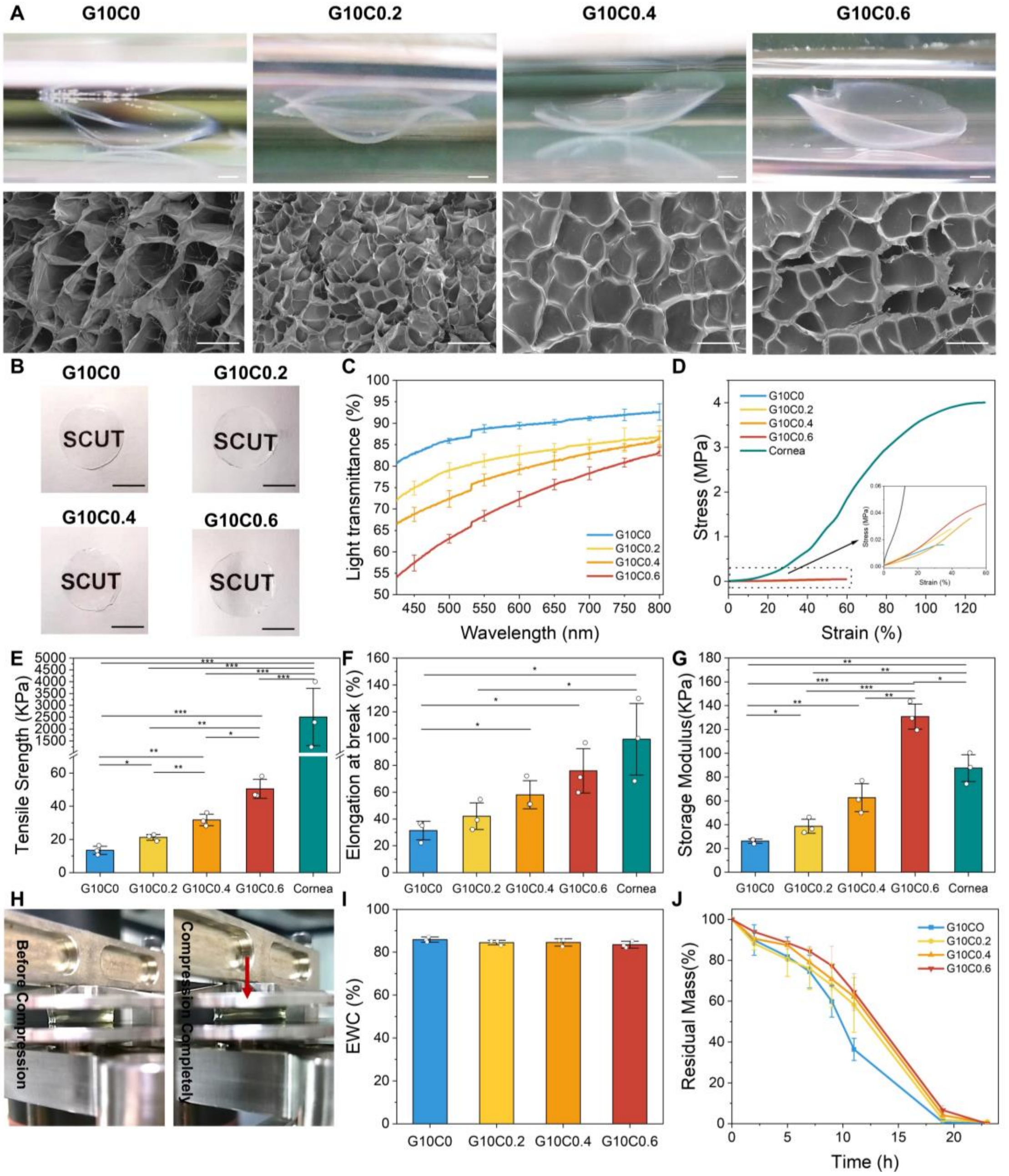

Figure S7. Properties of 3D printed cornea implants with different composition. A) The macroscopic (in water) and microscopic morphology of the printed cornea of G10C0, G10C0.2, G10C0.4, G10C0.6. The scale bar for macroscopic images were 1mm, for the SEM images were 100 μm. B) Digital photos of 3D printed cornea implants in a showing transparency. C) Transmittance curve of the samples in a after immersed in PBS for 2 hours. D) Strain–stress curves of the samples with different composition and native cornea. E-F) Mechanical properties of the samples in (D), tensile stress (E) and elongation at break (F) were calculated from data of strain–stress curves in (D), and histogram of compressive modulus (G) was acquired with ramp strain pattern, the storage modulus of cornea group was from reference[2]. H) showed the compressive force exerted to samples without destruction. I) Equilibrium water content diagram of the samples in (A) when immersed in PBS. J) Degradation rate curve of the samples in a when incubated in type I collagenase solution. All the results were calculated as mean ± standard deviation (SD) (n=3, biologically independent samples).

Physiochemical Characterization of Implants with Different Composition

Collagen addition to inks not only enhance printability but also influence material physiochemical properties, including the inner structure, light transmittance, tensile and compressive strength, water content and resistance to enzyme of implants with different ratios of GelMA to collagen, which can verify the optimal composition for generating implants with properties most similar to native corneal tissue. The GelMA/collagen implants having good curvature were observed when immersed in water with porous structures, which became denser with the increase of collagen, as shown in Figure S7A. From Figure S7B, we can see the implants were transparent and can be operated easily because of their soft properties. Light transmittance of implants with different composition were determined by UV-Vis spectrophotometer in the range of 430 to 800 nm after immersing into PBS for 2h (Figure S7C). Collagen addition decreased light transmittance overall, but it was still nearly 80% at 750 nm, which was similar with native cornea tissue, rising with the increase of wavelength and reaching 85% at 700 nm[3]. The tensile strain–stress curves of implants with different ratios were shown in Figure S7D, which behaved similar typical hard and brittle yield as native cornea. The tensile strength improved as the collagen concentration increased, and G10C0.6 possessed tensile strength of 51kPa, which was nearly three times of pure GelMA in Figure S7E. Though all implants presented brittle plastic characteristics close to native cornea[2], all implants had high elongation at break reaching about 80% of G10C0.6, indicating they can bear stretch to some extent. Figure S7G shows the compressive modulus of different composition of GelMA/collagen implants, and the addition of collagen can dramatically enhance the compressive modulus of implants. The compressive modulus of the native cornea tissue was 80 kPa, in our study, the storage modulus of G10C0.6 can reach 130kPa with the force imposed from both sides in Figure S7H without any destruction. The stretch and compressive experiments showed that the more the collagen was, the better mechanical properties they would acquire, which was consistent with the porous structures, as denser pores would result in complex network between GelMA and collagen as described in Figure S1B. Compared with native cornea, the printed cornea was not strong enough to bear stretch, its tensile strength was low though it behaved similar hard and brittle yield with native cornea, so in our animal model, we chose to use overlying sutures, the tensile strength still need to be improved further. But the

storage modulus was close or even more than native cornea, indicating it can bear corneal pressure completely.

Swelling ability of implants can manifest the degree of hydrophilicity and is a crucial feature of cornea, which is influenced by pore size. The measured water content of various implants showed no significant difference with pure GelMA of minor higher degree about 86%, all of which were over 80% similar to native cornea[4] as in Figure S7I. Resistance of enzymatic properties shown in Figure S7J reflected in suit stability to some extent, pure GelMA implants degraded completely within 20h while G10C0.6 at about 24h, the residual time was longer with the increase of collagen addition, this was partially due to collagen processed large molecules, which would set a barrier for enzyme to disintegrate molecular chains.

Based on high requirements of transparency and mechanical properties (Figure S7), the G10C0.6 implants with porous structures (Figure S7A), light transmittance of 83% at wavelength of 750 nm, tensile strength of 51 kPa, compressive modulus of 130 kPa, water content of 83% and mass enzymatic resistance were most similar to native cornea tissue. And they can be operated easily suitable for our following experiments (Figure S6G).

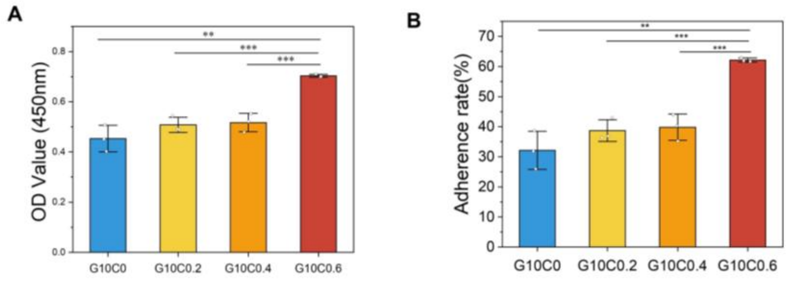

Figure S8. Adhesion results of different composition. A) CCK8 analysis of RCECs cultured on convex membranes after 6 h. B) Adherence rate histogram of the samples in (A). All the results were calculated as mean ± SD (n=3, biologically independent samples).

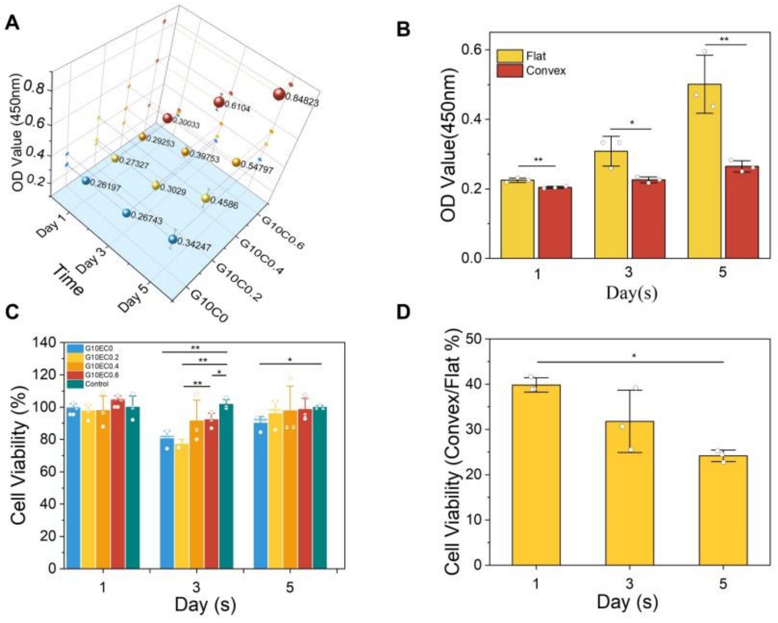

Figure S9. Comparison of cell proliferation between flat and convex implants. A) Proliferation of RCECs on convex implants with composition of G10C0, G10C0.2, G10C0.4, G10C0.6 at days 1, 3, 5. B) Proliferation between flat and convex implants of G10C0.6 at days 1, 3, 5. C) Cell viability of RCECs on convex implants with composition of G10C0, G10C0.2, G10C0.4, G10C0.6 at days 1, 3, 5. RCECs cultured on cell culture plates as control group. D) Cell viability on convex versus flat hydrogels.

Convex Constructs Affect Cell Proliferation

To investigate cell compatibility of 3D printed cornea implants, RCECs were inoculated on convex constructs with different composition, the OD value using CCK-8 method was acquired to verify cell proliferation. From Figure S9A, we can see clearly that with the increase of collagen concentration, the OD value grew bigger, especially at day 5, the value of G10C0.6 was 0.85, which was nearly three times of G10C0, revealing collagen had better bioactivity promoting cell growth. On the other hand, the OD value increased as time went by at days 1, 3 and 5, displaying cells can proliferate on these bioactive materials without cytotoxicity. As GelMA was a gelatin derivative containing many arginine-glycine-aspartic acids (RGD), which was a cell adhesion sequence that mimicked cell adhesion proteins and can bind to integrins facilitating cell adhesion behavior[5], we then studied cell adherence rate of different implants as in Figure S8, and there was no significant difference between collagen concentration from 0 to 0.4%, and G10C0.6 presented about twice the adherence rate of pure GelMA, which corresponded to cell proliferation behavior in Figure S9A. From Figure S9C, we can see cell viability of all groups were over 75%, showing they did not have toxicity, and cell viability of

G10C0.6 group were over 90% through the experimental period, indicating its good biocompatibility. Also, when we compared cell viability between convex to flat implants (Figure S9D), it was corresponded with cell proliferation, cells on flat implants can proliferate fast. Judging from the cell proliferation and adherence rate of implants, it came to a conclusion that collagen addition can boost biocompatibility. Besides, considering the most suitable printing parameters suppressing step effect, temperature at 27.5 °C and composition of G10C0.6 was selected in the following *in vitro* and *in vivo* experiments.

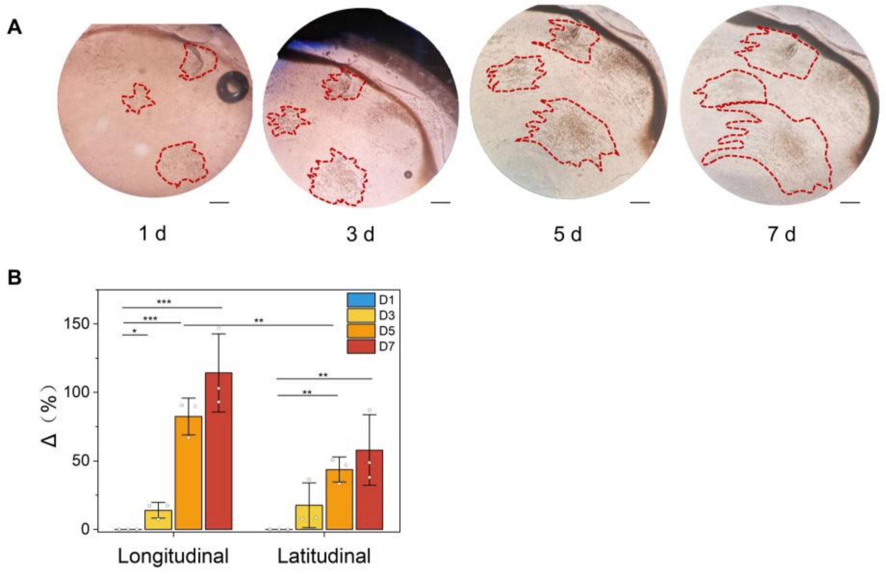

Figure S10. RECEs behavior on curvature surfaces. A) Bright field images to show cell growth trajectory when seeded on different parts of convex membrane at 1, 3, 5, 7 days, scale bar = 250 μm. B) Analysis of cell proliferation rate of the samples in a between latitudinal axis and longitudinal axis with time going by.

In our study, the growth rate between longitudinal axis and latitudinal axis were also calculated, on the whole, cells grew faster at longitudinal direction compared to latitudinal direction, even there was a reverse at day 3, the data showed no significant difference. And cells presented a rapid increase at day 5 at both directions, which may be connected to cell density and it reached cell logarithmic growth phase, displayed in Figure S10.

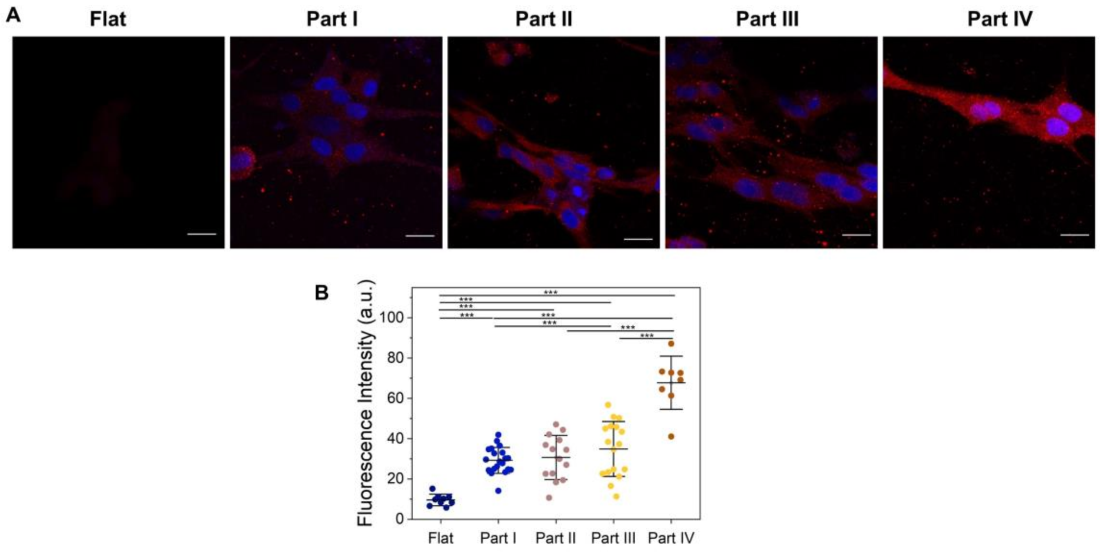

Figure S11. Slope gradient promoted cell adhesion. A) Representative fluorescence images of Vinculin immunostaining for RCECs after seeded for 5 days. (Scale bar = 25 μm). B) Quantification of Vinculin level of (A) using LAS AF Lite software.

The focal adhesion (FA) on the respective topography was investigated with a fluorescence staining of vinculin (focal adhesion protein), seen in Figure S11. By comparing the images qualitatively with one another, cells were finely spread with contracted actin stress fibers on all surfaces, and it is evident that more FAs were formed with the slope gradient became steeper, the fluorescence intensity of part IV was 126 a.u., nearly ten times of flat ones.

Table S2 qPCR primer sequences

Gene	Sequences 5'-3'
GAPDH-F	TTCAACAGCAACTCCCACTC
GAPDH-R	ATGTAGGCCATGAGGTCCAC
ITGA5-F	CTGGACTGGCAGAAGCAGAAGG
ITGA5-R	GGAGAAGTTGAGTGCGATGTGGAT
ITGB1-F	TGTCCAGTGCCGAGCCTTCAA
ITGB1-R	ACCAGCAGTCGTCCACATCCTT
ITGAv-F	TGGAGGACTGAGGTGAAGCAAGA
ITGAv-R	GCTACCAGGACCACCAAGAAGTAC
ITGB3-F	CAGTGGCAAGTCCATCCTGTATGTG
ITGB3-R	TTCGCTCTGGCTCGTTCTTCCT
ITGA6-F	TTCCTATCCTGATGTTGCTGTTGGT
ITGA6-R	AGGCTTTAACCTTGAGGCATATCCC
VCL-X2-F	GCAAAGCCATCCCTGACCTCAC
VCL-X2-R	CGGATCTGACTGAAGCATCTGAGC
FAK-F	AGCATCTATCCAGGTCAGGCATCTC
FAK-R	TGTCGGATTAGACGCTCTTCCATCA
RhoA-F	CTCATAGTCTTCAGCAAGGACC
RhoA-R	GGCGGTCATAATCTTCCTGTC
LmnA7-F	TCTCACAGCACGCACGCACTA
LmnA7-R	CGGTAAGTCAGCAAGGGATCATCTC

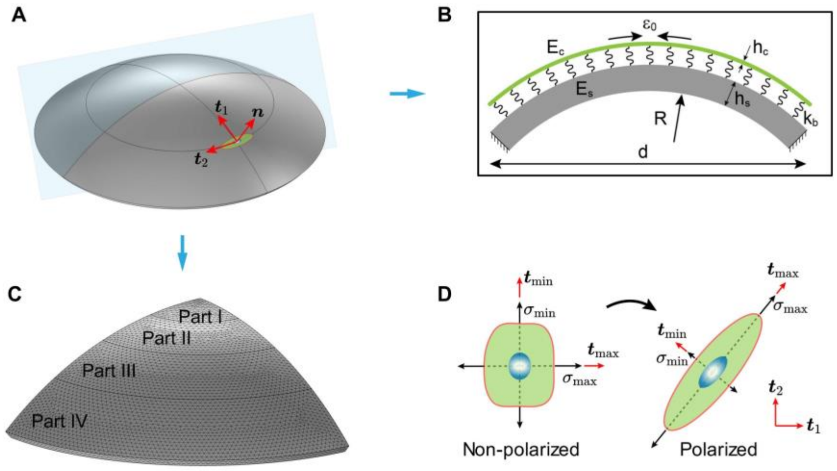

Figure S12. Finite element model (FEM) for predicting cell polarization and alignment on a 3D printed cornea. A) FEM of the cell layer and cornea substrate system. The local coordinate system of cell layer was defined by (t_1, t_2, n), representing two tangential directions (t_1 and t_2) and one normal direction (n). B) Schematic of the interaction between the cornea substrate and cell layer at the cross section of (A). The cornea substrate and cell layer were modeled as isotropic solid and pre-strained membrane, respectively, where cell layer was anchored on the cornea substrate via molecular bonds (treated as elastic springs k_b). C) The mesh setting of FEM and regions division. The tetrahedral solid mesh elements and triangular membrane mesh elements were used to discretize the cornea substrate and cell layer, respectively. For clarity, only a quarter of the mesh was shown. D) Schematic

illustration of cell polarization and alignment along the direction of the maximum principal stress in (t_1, t_2) tangent plane of cell layer.

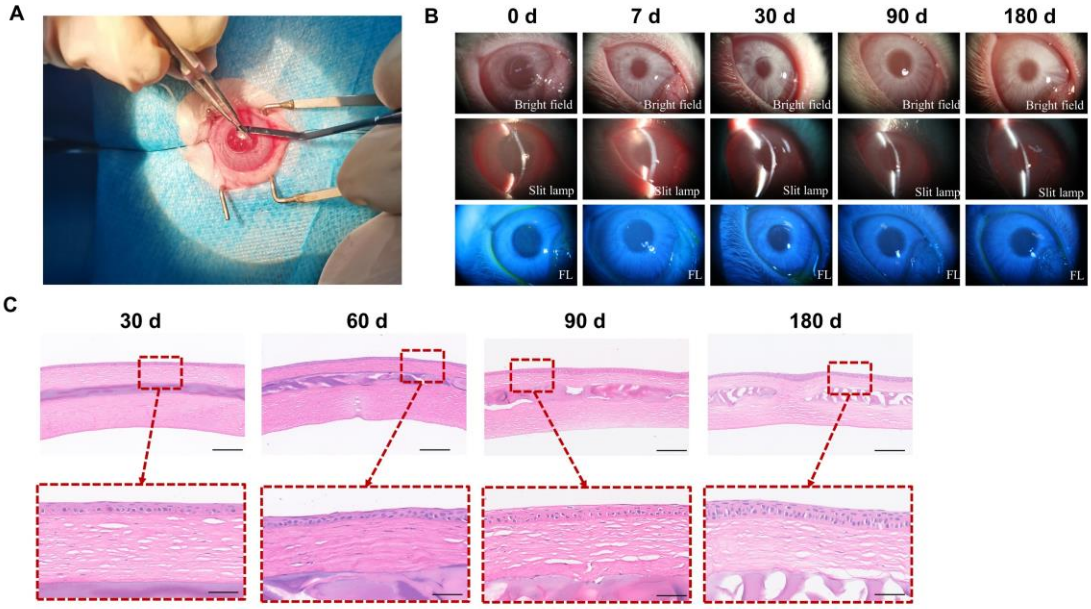

Figure S13. *In vivo* biocompatibility. A) Intrastromal corneal transplantation. B) Representative slit-lamp observations of cornea and images of corneal wound area stained with sodium fluorescein on day 0, day 7, day 30, day 90 and day 180. C) Post implantation histological examination (H&E staining) of corneas with different treatment after 30 days, 60 days 90 days and 180 days.

When conducting intrastromal corneal transplantation, we can see clearly that the printed corneal implants can be operated easily and keep complete during the whole procedure. After implanted for 180 days, the eye conditions of rabbits were well and transparent. From H&E staining, it was shown that materials kept constant without any degradation at 30 days, while it started to degrade at 90 days, the epithelial layer showed normal and healthy phenotype all the time and no inflammatory cells were detected. After half a year transplant, it showed that the adjacent tissue regenerated with the degradation of materials, which proved its biocompatibility and efficiency of promoting tissue regeneration. Altogether, our printed corneal implants showed good biocompatibility *in vivo*, providing basis for further animal experiment.

Experimental Section

High-throughput cornea printing apparatus: The custom-made bottom-up 3D pneumatic dispensing system (Regenovo, Hangzhou, China) was displayed in Figure 1A, which was comprised of a computer-controlled three-axis positioning stage, dual temperature-controlled extruders, a LED UV projector with 365nm irradiation wavelength and an optimized teflon

sprayed corneal platform. The projection area and pneumatic pressure that the apparatus can provide covers 100 convex corneal molds (290 mm x 110 mm) and 0-0.6 MPa, respectively. The temperature controller was equipped around the nozzle to ensure a homogeneous squeezed temperature, while a cooling system was also implemented to maintain the stable temperature after the deposition of GelMA/Collagen inks. The convex corneal mold was designed from average measurements of human using AutoCAD with diameter of 10 mm and curvature radius of 7.5 mm, and 100 molds were also made to facilitate high through-put printing. Convex not other structures[6] were chosen because natural cornea is thicker around the rim but thinner in the central, this structure was easier for ink to deposit and realize its complex structure. A STL file of cornea was converted to G-code utilizing Slic3r software and it was fixed in the boot interface of printing software (Regenovo) to service printing procedure. A concentric printing pattern was created from the bottom rim of cornel mold and then inwards and upwards towards the center, which can be shown in Figure1B.

GelMA Synthesis: High methacrylated gelatin was synthesised as previously described with minor variations[7]. Briefly, 10% (w/v) GelMA was prepared by dissolving 10 g gelatin powder (gel strength 240, purchased from Aladdin) in 100 mL phosphate buffered saline (PBS, Gibco) and allowing to dissolve completely for 1 h at 60 °C. 9 mL methacrylic anhydride (Aladdin) was then added at a rate of 200 μL/min and the reaction was maintained at 50 °C for 3 h. Once the substitution reaction was stopped by diluting the mixture (typically 5-10X) with PBS, the resulting solution would then be spun down at 5000 x g for 5 min to pellet unreacted MA and precipitated proteins. Thereafter, the solution was dialyzed at 40 °C for 7 days with a 12–14 kDa cut-off dialysis membrane (Yuanye Biotechnology Co., Ltd., Shanghai, China) to remove the remained low-molecular-weight impurities before lyophilized. ^{1}H-NMR spectra (in D_2O) were conducted on a Bruker Fourier 300 apparatus at room temperature to characterize the degree of methacrylation.

Preparation of GelMA/Collagen ink: A series of GelMA/Collagen inks were prepared by blending GelMA solution and collagen solution in different ratio with the addition of photoinitiator 2-hydroxy-4′-(2-hydroxyethoxy)-2-methylpropiophenone (I2959, Sigma-Aldrich; 0.5% (w/v) final concentration) and EDC (1-(3-Dimethylaminopropyl)-3-ethylcarbodiimide hydrochloride, Sigma-Aldrich) /NHS (N-hydroxysuccinimide, Sigma-

Aldrich). In short, GelMA was first resuspended in PBS and incubated in a 50 °C water bath until dissolved with I2959 final concentration of 0.5% (w/v), Collagen (type I, Pudao Lianxin Biotech, Co., Ltd., Guangzhou, China) was dissolved in 0.01 M HCl solution to generate collagen solution at 4 °C. A predetermined amount of GelMA/Collagen mixture was obtained by mixing these two components together at room temperature when the final concentration of GelMA was 5%(w/v), 10% (w/v), 15% (w/v) and collagen final concentration was 0.2% (w/v), 0.4% (w/v), 0.6% (w/v) permutably. Notably, both precursor solution should be high concentration in case it would be diluted after mixing, as their solution state was in conflict while GelMA was in gel situation at low temperature and collagen may lose activity at high temperature. Finally, 15 mg/mL crosslinking agent (EDC/NHS) solution was dripped to the mixture according to a mass ratio of collagen: crosslinking agent = 6:1 at room temperature, and they were stored at 4 °C under dark circumstance with centrifugation to eliminate bubbles before use.

HAMA Synthesis: HAMA was synthesized based on a method reported[8]. 2 g sodium hyaluronate (400-1000 kDa, Yuanye Biotechnology Co., Ltd., Shanghai, China) was dissolved in 1XPBS (200 mL) overnight. 7.5 mL of methacrylic anhydride was added dropwise with fast stirring. The pH of the mixture was maintained at 8.5 by adding 5M NaOH and the solution was stirred at room temperature for 12 h. The product was purified by 12–14 kDa cut-off dialysis membrane (Yuanye Biotechnology Co., Ltd., Shanghai, China) for 7days at room temperature before lyophilized (Martin Christ, ALPHA 2-4 LD plus).

Preparation of inks used for universality studies: Gelatin/Alginate inks were prepared with the ratio of 10%/0.5% (w/v) under 60°C, GelMA/Laponite inks with the ratio of 10%/0.5% (w/v) and GelMA/HAMA inks with the ratio of 10%/2% (w/v), the GelMA system would also add I2959 of 0.5% (w/v) to trigger the action. All inks were centrifuged to remove bubbles before use.

Rheology: The rheological properties[9] of different inks were evaluated by rheometer (MCR302, Anton Paar, Austria) by a plate–plate geometry with diameter of 25 mm. Firstly, post-gel inks were placed on the plate to completely fill the gap (size of 1 mm) between two plates. The measurements of viscosity were performed by varying the shear rate from 1-100 s^{-1} with temperature of 25 °C. And the sol-gel curves were measured when temperature ranging

from 45 °C to 0 °C at the rate of 4°C/min, with a constant frequency of 10 rad/s and a constant strain of 1%.

Physiochemical characterization: The mechanical properties of different composites of GelMA/Collagen implants were measured with a uniaxial load test machine (Instron 5967, USA). Tensile samples with rectangular shape (15 mm x 6 mm x 0.5 mm) were prepared in a rectangular mold with stress-strain sweeping of 0.25 N/min. Compressive samples with a diameter of 5mm and a thickness of 10mm were made to bear ramp strain at -50.0000 %/min to -100.00%. The maximum tensile strength, elongation at break, and the compressive modulus were recorded and calculated with the strain–stress tensile curves plotted. Three samples were measured for each composite to calculate the mean and standard deviation.

Transmittance test was conducted using UV-Vis spectrophotometer (UV3802, Shanghai UNICO, China) in the range of 430 nm–800 nm. Equilibrium water content (EWC) was measured before (lyophilized printed samples, w_0) and after (swelling equilibrium printed samples, w_t) the PBS (pH=7.4) swelling process which last for 6h, and it can be calculated as the equation of EWC (%) = (w_t -w_0)/ w_t x100%. The resistance of different samples to collagenase was evaluated as described previous[10]. Briefly, samples weighed 100mg were equilibrated in PBS for 1h and recorded the original weight (m_0), then they were immersed in collagenase type I solution (5 U/mL, Yuanye Biotechnology Co., Ltd., China) at 37°C for 24h, which need to change every 8 h to maintain the collagenase activity, samples were taken out to weigh at specified time with filter paper gently wiping off excess liquid, the weight was written as m_t, and the residual mass of samples was calculated by dividing m_t by m_0.

Roughness analysis: The roughness of different samples was measured by roughness instrument (W912C, Jenoptik, Germany) with probe type of TKU400. Put samples onto the pedestal of instrument and ensure their tight adhesion with each other, set λ_c of 0.80 mm and speed of 0.5 mm/s, after zero correction was complete, the measurement started and roughness and outline data were acquired within 1 minute.

Cell proliferation and adhesion analysis: RCECs were maintained in DMEM (Gibco) with 10% fetal bovine serum (FBS, Gibco) before use. 3D printed cornea implants with different ratios were sterilized by being immersed in 75% alcohol overnight and put into PBS solution for 1h to wash off remained alcohol. Cells were trypsinized and centrifuged to be counted while

suspended in the media until reached 90% confluency. For cell proliferation, 20 μL medium containing 2 x 10^4 cells were seeded dropwise onto each sample in the 24-well tissue culture plates, then they were maintained in a humidified environment at 37 °C and 5 % CO_2 for 2 h after seeding, after that another 480 μL medium was added gently into each well ensuring more cells adhering to materials. Media was changed every two days. At the time of 1,3 and 5 days after inoculation, CCK-8 kit (Dojindo, Kumamoto, Japan) was applied to characterize cell proliferation. 200 μL CCK-8 solution with 10% (v/v) concentration was added into each well when media was removed completely by PBS, after incubating at 37 °C and 5 % CO_2 for 2h in the dark circumstance, the optical density (OD) value at 450 nm was acquired with a micro-plate reader (Thermo 3001, USA). For cell adhesion rate, the procedure was similar as abovementioned, except the cell intensity was 2 x 10^4 on each material and culture time was 6h, besides standard adhesion curve was obtained by seeding different numbers of cells on the 24-well tissue culture plates, adhesion rate of series of composites was converted through standard adhesion curve.

Finite element model (FEM): The "Structural Mechanics Module" of the commercial finite element software COMSOL Multiphysics (Version 5.6; COMSOL, 2020) was adopted in the calculation of stress fields in cell layer adhering on the 3D printed cornea substrate. The geometry of the cornea is a spherical crown with a base diameter of $d = 10\ \mathrm{mm}$ and a spherical radius of $R = 7.5\ \mathrm{mm}$ (Figure S11 A-B). The cell layer and cornea substrate system were modeled as a pre-strained elastic membrane (Young's modulus was $E_c = 50\ \mathrm{kPa}$ and Poisson's ratio was $v_c = 0.45$)[11] adhering on elastic substrate (Young's modulus was $E_s = 50\ \mathrm{kPa}$ and Poisson's ratio was $v_s = 0.4$). The thickness of the cornea substrate and cell layer were set as $h_s = 200\ \mathrm{\mu m}$ and $h_c = 2\ \mathrm{\mu m}$ [11], respectively. The tetrahedral solid mesh elements and triangular membrane mesh elements were respectively used to discretize the cornea substrate and cell layer with a smallest mesh element size of $1\ \mathrm{\mu m}$ (Figure S11C). An initial strain $\varepsilon_0 = -0.1$ is set to the tangent plane of cell layer to model the self-contractility of cytoskeleton[11a,12]. As shown in Figure S11B, the cell layer is connected with substrate via adhesion molecules (treated as elastic springs) with areal stiffness ρk_b, where $k_b = 0.005\ \mathrm{nN/\mu m}$ is spring constants of adhesion molecule bond and $\rho = \rho_0 \exp(E_s/E_0)$ is the density of adhesion molecules associated with substrate stiffness and estimated from focal

adhesion area[11d,13]. Here the fitting parameters are $\rho_0 = 0.07\ \mu\text{m}^{-2}$ and $E_0 = 14.5$ kPa[11d,13]. The cell-substrate connection can be set through "Embedded Reinforcement" in "Multiphysics Couplings" module of COMSOL. The substrate is fixed at the edge, and the cell layer edge has free boundary condition (Figure S11B).

Theory of stress driven cell polarization and alignment: As shown in Figure S11D, at any point within the $(\boldsymbol{t}_1, \boldsymbol{t}_2)$ tangent plane of cell layer, the intercellular in-plane principal stresses, $\sigma_{\max}$ and $\sigma_{\min}$ $(\sigma_{\max} \geq \sigma_{\min})$ are defined in principal frame $(\boldsymbol{t}_{\max}, \boldsymbol{t}_{\min})$, in which shear stresses vanish and $\sigma_{\max}$ and $\sigma_{\min}$ are respectively called maximum and minimum principal stresses. Correspondingly, $\boldsymbol{t}_{\max}$ and $\boldsymbol{t}_{\min}$ are called principal orientations. Mechanically, the cytoskeleton mainly sustains tensile stress, but hardly bears shear stress. Thus, shear stress in cells will induce rotation of the cytoskeleton to align along the direction of maximum principal stress (tensile stress), where the shear stresses vanish[11b,11d]. Due to the chemical-mechanical coupling of cell polarization, cells are stretched along the maximum principal stress direction in cell layer and polarize along the same direction[11b,11d]. When the two principal stresses are isotropic, the cells are non-polarized with random orientations, otherwise, the cells are polarized and well-aligned when principal stresses are anisotropic (Figure S11D)[11b,11d]. To characterize the anisotropy of the principal stresses in cell layer, the maximum shear stress in tangent plane, defined as $\tau_{\max} = (\sigma_{\max} - \sigma_{\min})/2$, is introduced. It has been shown that the maximum shear stress drives cell polarization and alignment, and the cell polarization degree, i.e., cell aspect ratio, is proportional to the maximum shear stress[11b,11d]. That is, a larger maximum shear stress causes cells polarizing and aligning along the direction of the maximum principal stress with larger aspect ratio[11b-d,14].

Statistical Analysis: The data presented herein are expressed as mean ± standard deviation (SD) unless stated otherwise. Each experiment was repeated at least three times using biologically independent samples. Statistical analysis was performed using one-way ANOVA with multiple comparison analysis using Tukey's post hoc test, and p-values < 0.05 were considered statistically significant (*p < 0.05; **p < 0.01; ***p < 0.001). All statistical tests and graphs were generated using Origin 9.0 software.

References

[1] A. J. Seymour, S. Shin, S. C. Heilshorn, *Adv. Healthcare Mater.* **2021**, 10, e2100644.

[2] B. Kong, Y. Chen, R. Liu, X. Liu, C. Liu, Z. Shao, L. Xiong, X. Liu, W. Sun, S. Mi, *Nat. Commun.* **2020**, 11, 1435.

[3] J. D. Mallet, P. J. Rochette, *Photochem. Photobiol. Sci.* **2013**, 12, 1310.

[4] K. Tonsomboon, M. L. Oyen, *J. Mech. Behav. Biomed. Mater.* **2013**, 21, 185.

[5] E. Ruoslahti, M. Pierschbacher, *Science* **1987**, 238, 491.

[6] A. Isaacson, S. Swioklo, C. J. Connon, *Exp. Eye Res.* **2018**, 173, 188.

[7] A. I. Van den Bulcke, B. Bogdanov, N. De Rooze, E. H. Schacht, M. Cornelissen, H. Berghmans, *Biomacromolecules* **2000**, 1, 31.

[8] Y. Fan, Z. Yue, E. Lucarelli, G. G. Wallace, *Adv. Healthcare Mater.* **2020**, e2001410.

[9] J. Yin, M. Yan, Y. Wang, J. Fu, H. Suo, *ACS Appl. Mater. Interfaces* **2018**, 10, 6849.

[10] C. Deng, F. Li, J. M. Hackett, S. H. Chaudhry, F. N. Toll, B. Toye, W. Hodge, M. Griffith, *Acta Biomater.* **2010**, 6, 187.

[11] a)S. He, Y. Su, B. Ji, H. Gao, *J. Mech. Phys. Solids* **2014**, 70, 116; b)S. He, C. Liu, X. Li, S. Ma, B. Huo, B. Ji, *Biophys. J.* **2015**, 109, 489; c)C. Liu, J. Xu, S. He, W. Zhang, H. Li, B. Huo, B. Ji, *J. Mech. Behav. Biomed. Mater.* **2018**, 88, 330; d)S. He, Y. Green, N. Saeidi, X. Li, J. J. Fredberg, B. Ji, L. M. Pismen, *J. Mech. Phys. Solids* **2020**, 137, 103860.

[12] S. Deguchi, T. Ohashi, M. Sato, *J. Biomech.* **2006**, 39, 2603.

[13] J. M. Goffin, P. Pittet, G. Csucs, J. W. Lussi, J.-J. Meister, B. Hinz, *J. Cell Biol.* **2006**, 172, 259.

[14] a)D. T. Tambe, C. Corey Hardin, T. E. Angelini, K. Rajendran, C. Y. Park, X. Serra-Picamal, E. H. Zhou, M. H. Zaman, J. P. Butler, D. A. Weitz, *Nat. Mater.* **2011**, 10, 469; b)C. Liu, S. He, X. Li, B. Huo, B. Ji, *J. Appl. Mech.* **2016**, 83, 051014.

Supporting Movie Captions

Movie S1. Printing cornea procedure.

Supporting\Movie S1.mp4

Movie S2. Flat nuclei deformation.

Supporting\Movie S2 .mp4

Movie S3. Part IV nuclei deformation.

Supporting\Movie S3 .mp4
